# Gender Disparities in the Association Between Educational Attainment and Cardiovascular-Kidney-Metabolic Syndrome: Cross-Sectional Study

**DOI:** 10.2196/57920

**Published:** 2024-08-23

**Authors:** Yi Ding, Xianglin Wu, Qiuyu Cao, Jiaojiao Huang, Xiaoli Xu, Youjin Jiang, Yanan Huo, Qin Wan, Yingfen Qin, Ruying Hu, Lixin Shi, Qing Su, Xuefeng Yu, Li Yan, Guijun Qin, Xulei Tang, Gang Chen, Min Xu, Tiange Wang, Zhiyun Zhao, Zhengnan Gao, Guixia Wang, Feixia Shen, Zuojie Luo, Li Chen, Qiang Li, Zhen Ye, Yinfei Zhang, Chao Liu, Youmin Wang, Tao Yang, Huacong Deng, Lulu Chen, Tianshu Zeng, Jiajun Zhao, Yiming Mu, Shengli Wu, Yuhong Chen, Jieli Lu, Weiqing Wang, Guang Ning, Yu Xu, Yufang Bi, Mian Li

**Affiliations:** 1 Department of Endocrine and Metabolic Diseases, Shanghai Institute of Endocrine and Metabolic Diseases, Shanghai Jiao Tong University School of Medicine Affiliated Ruijin Hospital, Shanghai, China; 2 Jiangxi Provincial People’s Hospital Affiliated to Nanchang University, Nanchang, China; 3 The Affiliated Hospital of Southwest Medical University, Luzhou, China; 4 The First Affiliated Hospital of Guangxi Medical University, Nanning, China; 5 Zhejiang Provincial Center for Disease Control and Prevention, Hangzhou, China; 6 Affiliated Hospital of Guiyang Medical College, Guiyang, China; 7 Xinhua Hospital Affiliated to Shanghai Jiaotong University School of Medicine, Shanghai, China; 8 Tongji Hospital, Tongji Medical College, Huazhong University of Science and Technology, Wuhan, China; 9 Sun Yat-sen Memorial Hospital, Sun Yat-sen University, Guangzhou, China; 10 The First Affiliated Hospital of Zhengzhou University, Zhengzhou, China; 11 The First Hospital of Lanzhou University, Lanzhou, China; 12 Fujian Provincial Hospital, Fujian Medical University, Fuzhou, China; 13 Dalian Municipal Central Hospital, Dalian, China; 14 The First Hospital of Jilin University, Changchun, China; 15 The First Affiliated Hospital of Wenzhou Medical University, Wenzhou, China; 16 Qilu Hospital of Shandong University, Jinan, China; 17 The Second Affiliated Hospital of Harbin Medical University, Harbin, China; 18 Central Hospital of Shanghai Jiading District, Shanghai, China; 19 Jiangsu Province Hospital on Integration of Chinese and Western Medicine, Nanjing, China; 20 The First Affiliated Hospital of Anhui Medical University, Hefei, China; 21 The First Affiliated Hospital of Nanjing Medical University, Nanjing, China; 22 The First Affiliated Hospital of Chongqing Medical University, Chongqing, China; 23 Union Hospital, Tongji Medical College, Huazhong University of Science and Technology, Wuhan, China; 24 Shandong Provincial Hospital affiliated to Shandong University, Jinan, China; 25 Chinese People’s Liberation Army General Hospital, Beijing, China; 26 Karamay Municipal People’s Hospital, Karamay, China

**Keywords:** cardiovascular-kidney-metabolic syndrome, education, health behavior, sex difference, cross-sectional study, gender

## Abstract

**Background:**

Cardiovascular-kidney-metabolic (CKM) health is affected by social determinants of health, especially education. CKM syndrome has not been evaluated in Chinese population, and the association of education with CKM syndrome in different sexes and its intertwined relation with lifestyles have not been explored.

**Objective:**

We aimed to explore the association between educational attainment and the prevalence of CKM syndrome stages in middle-aged and older Chinese men and women as well as the potential role of health behavior based on Life’s Essential 8 construct.

**Methods:**

This study used data from the nationwide, community-based REACTION (Risk Evaluation of Cancers in Chinese diabetic individuals: a longitudinal study). A total of 132,085 participants with complete information to determine CKM syndrome stage and education level were included. Educational attainment was assessed by the self-reported highest educational level achieved by the participants and recategorized as low (elementary school or no formal education) or high (middle school, high school, technical school/college, or above). CKM syndrome was ascertained and classified into 5 stages according to the American Heart Association presidential advisory released in 2023.

**Results:**

Among 132,085 participants (mean age 56.95, SD 9.19 years; n=86,675, 65.62% women) included, most had moderate-risk CKM syndrome (stages 1 and 2), and a lower proportion were at higher risk of CKM (stages 3 and 4). Along the CKM continuum, low education was associated with 34% increased odds of moderate-risk CKM syndrome for women (odds ratio 1.36, 95% CI 1.23-1.49) with a significant sex disparity, but was positively correlated with high-risk CKM for both sexes. The association between low education and high-risk CKM was more evident in women with poor health behavior but not in men, which was also interactive with and partly mediated by behavior.

**Conclusions:**

Low education was associated with adverse CKM health for both sexes but was especially detrimental to women. Such sex-specific educational disparity was closely correlated with health behavior but could not be completely attenuated by behavior modification. These findings highlight the disadvantage faced by women in CKM health ascribed to low education, underscoring the need for public health support to address this inequality.

## Introduction

Cardiovascular disease (CVD), chronic kidney disease (CKD), and diabetes have been a set of chronic diseases, which continuously burden human well-being and health systems globally, especially among low- or middle-income countries [1-4]. Growing concerns have been raised about the underlying pathophysiological interconnections they share [5] as well as the adverse consequences related to their confluency [6]. On the other hand, opportunities also emerged regarding several promising therapies that showed metabolic, renal, and cardiovascular benefits [7,8]. The American Heart Association (AHA) has thus proposed a conceptual framework of cardiovascular-kidney-metabolic (CKM) syndrome [9] to provide a holistic comprehension, prevention strategies, and management approaches for the CKM continuum.

However, CKM health is determined by the interplay of biological predisposition and social determinants of health (SDOH). Education has been recognized as a predominant SDOH due its profound and consistent impact on health outcomes. Poor education at the individual level and educational inequalities at the community level on health outcomes significantly affect health, including various CKM components [10-16] and mortality [13,17]. Additionally, education has the largest average marginal effect among all socioeconomic factors [18]. Nevertheless, most studies lacked representation for populations with a poorer educational background, and a few of them covered the full spectrum of CKM disorders. Furthermore, evidence suggested that CVD risk associated with poor education varied significantly between sexes [19], mostly to the detriment of women, but differential gender vulnerability to progression along the CKM spectrum conferred by educational attainment remains a major knowledge gap. The underlying mechanisms underpinning these differences were also unclear, and some intermediate factors, especially lifestyle, may have a potential role in addressing sex-specific educational inequalities. The Life’s Essential 8 (LE8) construct, covering both health behavior and health factor metrics, has been recommended by the AHA as a holistic framework for achieving and monitoring CKM health. It is, therefore, important to reveal the different associations between education and CKM stages in men and women as well as the intertwined relations with potential mediators, especially health behavior assessed by the LE8, which may shape the differences. It would provide gender-based, socioeconomic factor–incorporated intervention targets for both individuals and the public health system to improve CKM health.

Therefore, in a large-scale community-based cohort across mainland China, by comprehensively evaluating the burden of CKM syndromes, we aimed to explore how educational attainment differently shaped the susceptibility to different CKM stages in men and women, with a focus on the role of health behaviors as a potential mediator based on the LE8 construct.

## Methods

### Study Population

REACTION (Risk Evaluation of Cancers in Chinese Diabetic Individuals: a longitudinal study) was a nationwide community-based cohort study, which has been previously described elsewhere [20]. The baseline phase was conducted from 2011 to 2012. A total of 25,9657 adults were recruited from 25 communities covering 16 provinces across mainland China. For this study, we excluded participants with insufficient data to determine CKM stages (n=126,876) or missing information on education (n=696). Ultimately, 132,085 people were included (Figure S1 in Multimedia Appendix 1). Major baseline characteristics between the participants recruited and those excluded were generally similar (Table S1 in Multimedia Appendix 1).

### Ethical Considerations

The study was approved by the Medical Ethics Committee of Rui-jin Hospital [approval number: (2011)临伦审第(14)号], and written informed consent was collected from all participants.

### Data Collection

Baseline examinations were conducted through face-to-face interviews along with a structured questionnaire, anthropometric measurements, and blood sampling at local community clinics. Educational attainment was assessed by the self-reported highest educational level achieved by the participants and recategorized as low (elementary school or no formal education) or high (middle school, high school, technical school/college, or above). Other socioeconomic factors, including marital status, living status, and occupation were also recorded through the questionnaire. Detailed information is presented in Multimedia Appendix 1.

### Definition of CKM Syndrome Stages

CKM syndrome was classified into 5 stages according to a presidential advisory proposed by the AHA [9] in 2023, as displayed in Table S2 in Multimedia Appendix 1. Specifically, stage 0 was defined as the absence of any CKM risk factors; stage 1 was defined as having excess body weight, abdominal obesity, or dysfunctional adipose tissue (manifest as prediabetes), without the presence of other metabolic risk factors or CKD; stage 2 was defined as the presence of metabolic risk factors or moderate- to high-risk CKD; stage 3 was defined as risk equivalents of subclinical CVD (eg, very-high-risk CKD or a high predicted 10-year CVD risk based on the AHA predicting risk of cardiovascular disease events (PREVENT) equations [21]); and stage 4 was defined as clinical CVD, including coronary heart disease, myocadiac infarction, stroke, or peripheral artery disease. Following the AHA’s scientific statement [5], we combined CKM stages 1 and 2 to represent borderline to intermediate predicted CVD risk, and CKM stages 3 and 4 to represent high predicted risk [22].

### Definition of Health Behavior and Health Factor Based on the LE8

The updated LE8 definition proposed by the AHA [23] was used to evaluate health behavior and the achievement of health factors. Health behaviors (eg, nicotine exposure, diet, physical activity, and sleep) were evaluated using a standardized questionnaire. Health factors were measured either in the study center (BMI, blood pressure, and plasma glucose level) or the central laboratory (blood lipids and glycated hemoglobin [HbA_1c_]). Each of the 8 metrics above was scored from 0 to 100, and each score of 80-100 was considered optimal. Achievement of overall optimal health behavior or health factors was reflected by an average score of 80-100 across 4 health behaviors or 4 factor metrics, respectively. We additionally calculated a health behavior score by the number of optimal health behaviors and recategorized it into 3 groups (0-1 optimal health behavior; 2 optimal health behaviors; and 3-4 optimal health behaviors). Detailed methods and criteria were presented in Table S3 in Multimedia Appendix 1.

### Statistical Analysis

The analyses are detailed in Multimedia Appendix 1. First, to investigate the association between educational attainment and CKM outcomes, logistic regressions were conducted to estimate odds ratios (ORs) and 95% CIs for the association of low education with moderate-risk CKM (stage 1 to 2) and high-risk CKM (stage 3 to 4). In the total population, the interaction term between education level and sex was added to obtain the *P* value for interaction and the women-to-men ratio of odds ratio (ROR) [24]. Next, to further compare the condition of both LE8 domains across different CKM stages within each sex and education strata, we estimated age-adjusted proportions (and 95% CIs) of participants who achieved optimal LE8 health behavior and factors according to the CKM stage.

To explore the sex-specific role of health behavior in the association between education level and high-risk CKM syndrome, we conducted the following: (1) stratified analysis according to behavior groups, (2) joint analysis by classifying participants according to the combination of education levels and behavior groups, and (3) mediation analysis of each component of LE8 health behavior and the overall score on the relation between education level and outcome.

Relative index of inequality (RII) was used to illustrate educational inequality [25] in high-risk CKM in the total population as well as among men and women. It could be interpreted as the relative increase of the prevalence of the outcome predicted for the hypothetical lowest versus highest end of the education continuum. Stepwise adjustments for socioeconomic and LE8 behavior factors were conducted to evaluate their contribution to educational inequalities in the outcome.

Several sensitivity analyses were performed. First, we repeated our analyses by subdividing the educational level into 4 categories (elementary school or below, middle school, high school, and college school or above), following the modified International Standard Classification of Education scale [26,27]. Second, we repeated our analyses in multiple imputation data sets imputed for missing baseline information and outcome (Table S10-S13 in Multimedia Appendix 1.

Statistical analyses were performed with SAS (version 9.4; SAS Institute) and R (version 4.3.3; The R Foundation). Statistical significance level was a 2-tailed *P* value of <.05.

## Results

As shown in Table 1, a total of 132,085 participants (mean age 56.95, SD 9.19 years) were included from the REACTION study, among whom 45,410 (34.38%) were men, and 86,675 (65.62%) were women. Compared with men, a greater proportion of women received low education, and they were more likely to be engaged in low-level occupations, unmarried, and living alone. In total, a vast majority of participants had moderate-risk CKM syndrome (stage 2: n=81,693, 61.85%; stage 1: n=27,559, 20.86%), and a low proportion had high-risk CKM syndrome (stage 3: n=8786, 6.65%; stage 4: n=8571, 6.49%), while an even smaller minority had low-risk CKM syndrome (stage 0: n=5476, 4.15%). Generally, women had a better distribution of CKM syndrome than men, with a higher proportion of low-to-moderate–risk stages, but a relatively smaller proportion of high-risk stages. Women with high education had consistently higher prevalence of stage 0 to 2 and a corresponding lower prevalence of stages 3 and 4 compared to their less educated counterparts (Figure S2 in Multimedia Appendix 1). However, CKM distribution disparities between high and low education among men were mainly concentrated on stage 3, while no evident differences were observed for the proportion of stages 0 and 1.

**Table 1.** Baseline characteristics of the study population stratified by sex.

Characteristics		Complete sample (N=132,085)	Men (n=45,410, 34.38%)	Women (n=86,675, 65.62%)
Age (years), mean (SD)		56.95 (9.19)	57.84 (9.50)	56.48 (8.98)
**Socioeconomic factors, n (%)**				
	Low education	40,234 (30.46)	10,739 (23.65)	29,495 (34.03)
	Low-level occupation^a^	40,105 (30.58)	12,032 (26.70)	28,073 (32.61)
	Unmarried	11,325 (8.59)	1824 (4.02)	9501 (10.98)
	Living alone	5272 (4.00)	1139 (2.51)	4133 (4.78)
**LE8^b^ health behaviors, n (%)**				
	Optimal nicotine exposure	100,097 (75.78)	16,029 (35.30)	84,068 (96.99)
	Optimal diet	27,752 (24.62)	8312 (21.86)	19,440 (26.02)
	Optimal physical activity	31,591 (24.55)	12,840 (29.08)	18,751 (22.18)
	Optimal sleep	90,087 (76.99)	30,296 (76.25)	59,791 (77.37)
**CKM^c^ syndrome staging factors, mean (SD)**				
	BMI (kg/m^2^)	24.60 (3.58)	24.76 (3.50)	24.52 (3.62)
	Waist circumference (cm)	84.36 (9.90)	86.86 (9.72)	83.06 (9.74)
	Systolic BP^d^ (mm Hg)	133.32 (21.03)	135.72 (20.35)	132.06 (21.27)
	Diastolic BP^d^ (mm Hg)	78.63 (11.16)	80.84 (11.33)	77.47 (10.90)
	Fasting glucose (mg/dL)	107.34 (29.58)	110.23 (32.26)	105.82 (27.96)
	Post-load glucose (mg/dL)	149.34 (69.53)	151.38 (75.18)	148.27 (66.36)
	HbA1c^e^ (%)	6.03 (1.04)	6.06 (1.12)	6.01 (0.99)
	Total cholesterol (mg/dL)	191.38 (44.37)	184.81 (42.66)	194.82 (44.86)
	LDL^f^ cholesterol (mg/dL)	110.62 (33.97)	107.04 (32.53)	112.49 (34.55)
	HDL^g^ cholesterol (mg/dL)	51.04 (13.74)	48.11 (13.90)	52.57 (13.41)
	Triglycerides (mg/dL)	117.84 (83.28, 171.88)	118.72 (83.28, 178.09)	117.84 (84.17, 169.23)
	Estimated GFR^h^ (mL/min/1.73 m^2^)	95.36 (86.27, 102.47)	95.93 (86.62, 103.02)	95.05 (86.10, 102.15)
	Urinary ACR^i^ (mg/g)	6.30 (3.80, 12.52)	5.38 (3.38, 10.51)	6.84 (4.11, 13.47)
**Metabolic disease, n (%)**				
	Diabetes	31,994 (24.22)	12,280 (27.04)	19,714 (22.74)
	Hypertension	55,543 (42.05)	21,315 (46.94)	34,228 (39.49)
	Hyperlipidemia	54,955 (41.61)	21,057 (46.37)	33,898 (39.11)
**Medication, n (%)**				
	Hypoglycemic drugs	9976 (7.55)	3928 (8.65)	6048 (6.98)
	Antihypertensive drugs	16,614 (12.60)	5833 (12.85)	10,811 (12.47)
	Lipid-lowering drugs	1118 (0.85)	377 (0.83)	741 (0.85)
**CKM syndrome stage, n (%)**				
	Stage 0	5476 (4.15)	1556 (3.43)	3920 (4.52)
	Stage 1	27,559 (20.86)	8180 (18.01)	19,379 (22.36)
	Stage 2	81,693 (61.85)	27,737 (61.08)	53,956 (62.25)
	Stage 3	8786 (6.65)	4732 (10.42)	4054 (4.68)
	Stage 4	8571 (6.49)	3205 (7.06)	5366 (6.19)

a Defined as farmer, housewife, or unemployed. Participants who self-reported that they were retired were not considered to be unemployed.

b LE8: Life’s Essential 8.

c CKM: cardiovascular-kidney-metabolic.

d BP: blood pressure.

e HbA_1c_: glycated hemoglobin.

f LDL: low-density lipoprotein.

g HDL: high-density lipoprotein.

h GFR: glomerular filtration rate.

i ACR: albumin-creatinine ratio.

### Association Between Low Education and Upgrading CKM Stages in Men and Women

Table 2 presented the sex-specific odds ratio of low education for moderate-risk and high-risk CKM syndrome. Compared with participants with high education, low education was associated with a 1.36-fold (95% CI 1.23-1.49) higher odds of moderate-risk CKM syndrome in women but not men, with a significant women-to-men ROR (2.45, 95% CI 2.13 to 2.83). However, low education was positively associated with high-risk CKM for both sexes, while no evident sex difference was found. After adjusting for LE8 behaviors, these associations remained consistent. Results were similar in sensitivity analyses (Table S7 and S10 in Multimedia Appendix 1).

**Table 2.** Sex-specific odds ratios (ORs) and ratio of odds ratios (RORs) for moderate-risk and high-risk cardiovascular-kidney-metabolic (CKM) syndrome attributable to low education. Model 1 was adjusted for age, study center, and other socioeconomic factors (including marital status, living status, and occupation). Model 2 was further adjusted for Life’s Essential 8 health behavior, including nicotine exposure, physical activity, diet, and sleep, as well as medication information, including hypoglycemic drugs, antihypertensive drugs and lipid-lowering drugs (used or not). For high-risk CKM syndrome, CKM stages 0 to 2 were used as reference; for moderate-risk CKM syndrome, CKM stage 0 was used as reference. OR was calculated by contrasting low education (elementary school or below) with high education (middle school or above) in men and women, respectively. In the total population, the interaction term between education and sex was added to obtain the *P *value for interaction and the women-to-men RORs.

Characteristics		Total cases, n(%)	Model 1				Model 2			
			MenOR(95% CI)	WomenOR(95% CI)	Women-to-men ROR	*P*_for interaction_	MenOR(95% CI)	WomenOR(95% CI)	Women-to-men ROR	*P*_for interaction_
**OR for the prevalence of moderate-risk CKM syndrome**										
	High education	77,694 (84.6)	1.00 (reference)	1.00 (reference)	—^a^	—	1.00 (reference)	1.00 (reference)	—	—
	Low education	31,558 (78.4)	0.87 (0.76, 1.00)	1.35 (1.23, 1.49)	2.52 (2.19, 2.91)	<.001	0.88 (0.77, 1.01)	1.36 (1.23, 1.49)	2.45 (2.13, 2.83)	<.001
**OR for the prevalence of high-risk CKM syndrome**										
	High education	9904 (10.8)	1.00 (reference)	1.00 (reference)	—	—	1.00 (reference)	1.00 (reference)	—	—
	Low education	7453 (18.5)	1.26 (1.18, 1.35)	1.25 (1.18, 1.33)	0.99 (0.92, 1.07)	.86	1.32 (1.23, 1.42)	1.29 (1.22, 1.37)	0.97 (0.89, 1.05)	.39

a Not applicable.

### Achievement of Optimal LE8 Health Targets Across the CKM Continuum Across Different Sex and Education Strata

**Figure 1.**
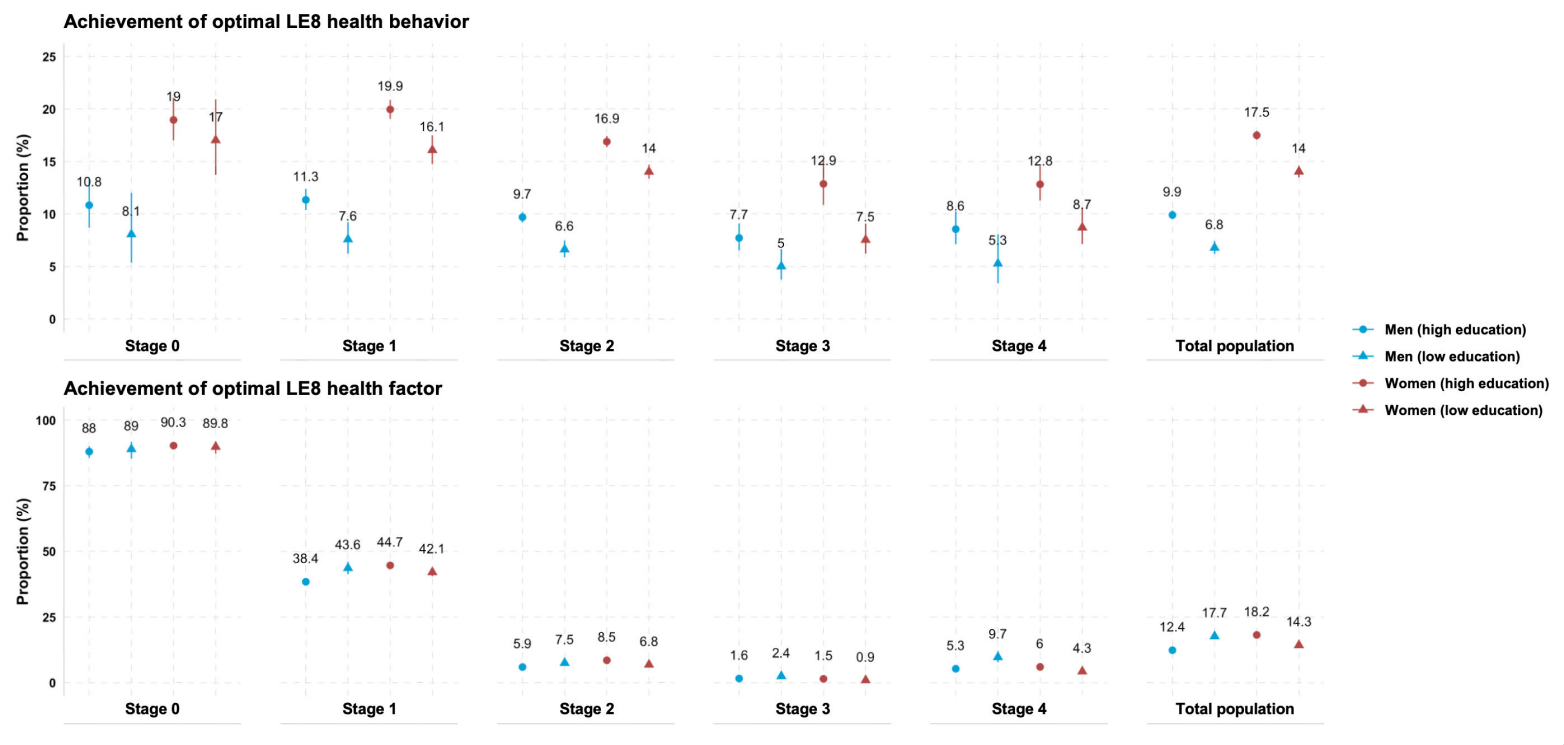
Achievement of optimal Life’s Essential 8 (LE8) health behavior and health factor targets in men and women with different education levels, according to different CKM stages. Analyses were conducted among participants with complete information on LE8 health behavior (n=99,124) or LE8 health factor (n=131,793). Age-adjusted estimates of proportions (and 95% CIs) were calculated using logistic regressions. A restricted cubic spline function was applied to age, with 4 knots placed at the fifth, 35th, 65th, and 95th percentiles.

We assessed the age-adjusted proportions of participants who achieved optimal health behavior and optimal health factors along the CKM spectrum across different sex and education strata (Figure 1; Table S4 in Multimedia Appendix 1). Women had generally better control of both domains than men. Substantially, a larger proportion of well-educated participants achieved optimal health behavior targets in both sexes. Regarding health factors, women with high education continued to exhibit better achievement of optimal health factors than their lesser educated counterparts, while the opposite was true for men. Notably, in high-risk CKM stages, women with low education had the worst achievement of overall health factor targets. Results were similar in the multiple imputation datasets (Table S11 in Multimedia Appendix 1).

### Interactive and Mediating Association of LE8 Health Behavior and Education Level With High-Risk CKM Syndrome in Men and Women

**Figure 2.**
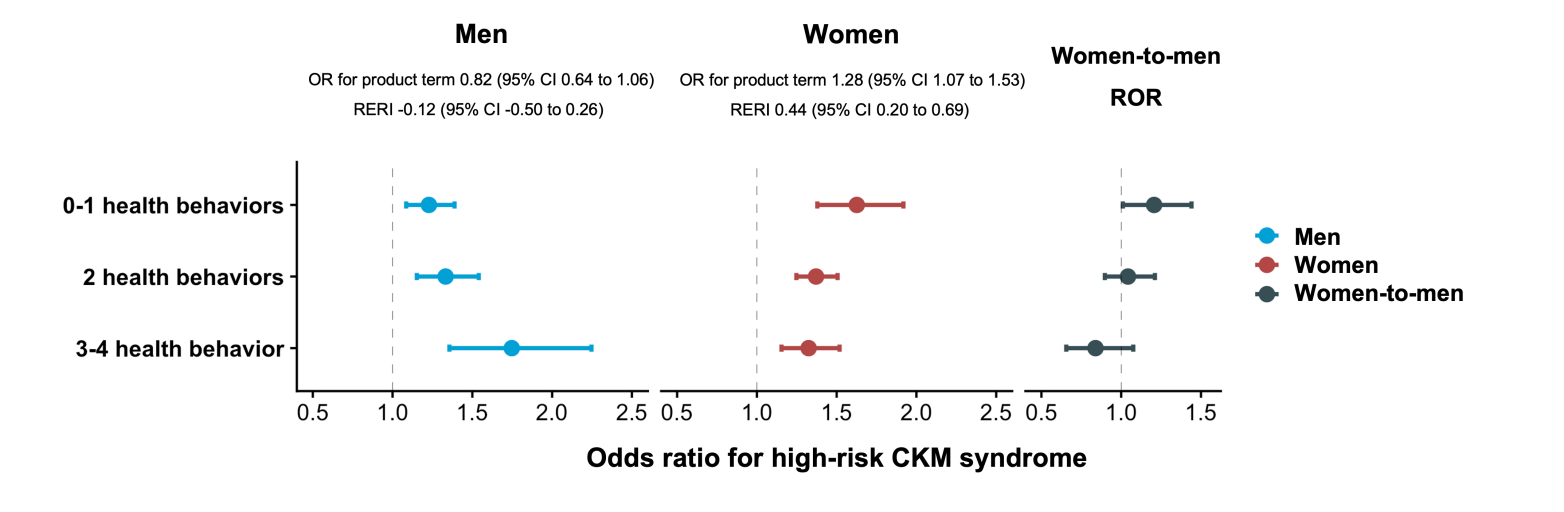
Association of low education with high-risk cardiovascular-kidney-metabolic (CKM) syndrome in men and women with different Life’s Essential 8 (LE8) health behaviors. Analyses were conducted among participants with complete information on LE8 health behaviors (n=99,124). Models were adjusted for age; study center; other socioeconomic factors, including marital status, living status, and occupation, as well as medication information, including hypoglycemic drugs, antihypertensive drugs, and lipid-lowering drugs (used or not). In each health behavior subgroup, those with high education (middle school or above) were selected as the control group. Multiplicative interaction was evaluated using odds ratios (ORs) for the product term between LE8 health behavior (0-1 optimal health behavior vs 3-4 optimal health behaviors) and education level (low vs high), and the multiplicative interaction was statistically significant when its CI did not include 1. Additive interaction was evaluated using relative excess risk due to interaction (RERI) between the LE8 health behavior and education level, and the additive interaction was statistically significant when its CI did not include 0. In the total population, the interaction term between education and sex was added to obtain the *P* value for interaction and the women-to-men ratio of odds ratios (ROR).

**Figure 3.**
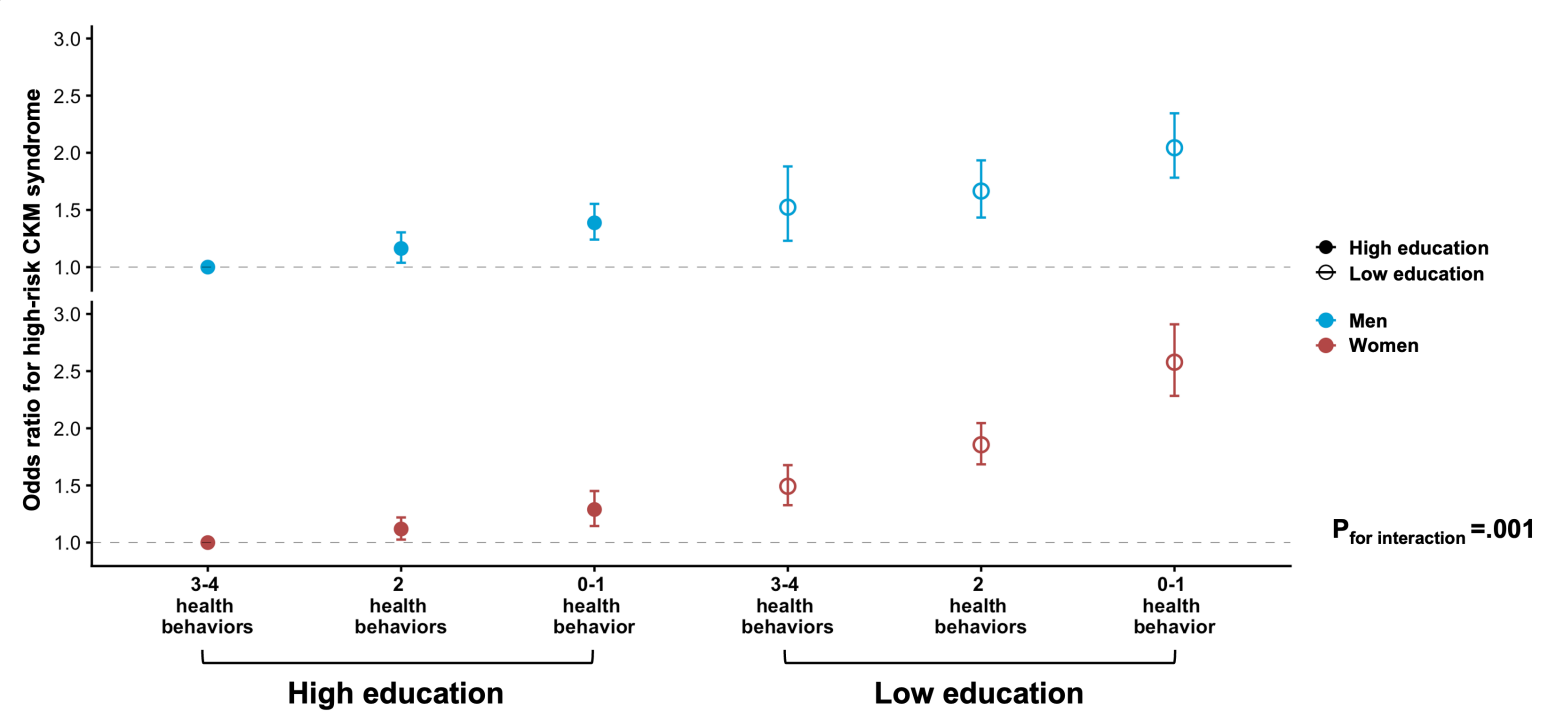
Joint associations of education level and Life’s Essential 8 (LE8) health behavior with high-risk cardiovascular-kidney-metabolic (CKM) syndrome in men and women. Analyses were conducted among participants with complete information on LE8 health behaviors (n=99,124). Models were adjusted for age; study center; other socioeconomic factors, including marital status, living status, and occupation, as well as medication information, including hypoglycemic drugs, antihypertensive drugs, and lipid-lowering drugs (used or not). The combination of high education (middle school or above) and 3-4 optimal health behaviors was selected for the control group. In the total population, the *P* value for interaction was obtained using the product term of combined education-behavior group (low education and 0-1 optimal health behavior vs high education and 3-4 optimal health behaviors) and sex.

As displayed in Figure 3 in Multimedia Appendix 1, the LE8 health behavior score increased with education level in both sexes, with generally higher scores in women. Notably, no significant interaction was found between health behavior groups and education level in high-risk CKM in men, whereas both multiplicative and additive interactions were observed in women (Figure 2 and Table S5 in Multimedia Appendix 1). Across the health behavior groups, the association between low education and high-risk CKM syndrome varied between sexes. In women with the least heath behavior, low education was associated with 63% (OR 1.63, 95% CI 1.38-1.92) higher odds of high-risk CKM, with a significant women-to-men ROR (1.21, 95% CI 1.01-1.44; *P*_for interaction_ =.04). With the improvement of health behavior, the detrimental association by low education was slightly attenuated for women but became more evident for men, and the sex differences were correspondingly narrowed. When assessing the joint associations, ORs for those with a combination of low education and 0-1 health behavior were 2.04 (95% CI 1.78-2.35) in men and 2.58 (95% CI 2.28-2.91) in women, with a significant sex difference (*P*_for interaction_=.001; Figure 3 and Table S6 in Multimedia Appendix 1). Results were not materially changed in sensitivity analyses (Tables S8, S9, S12, and S13 in Multimedia Appendix 1). The mediation proportion by suboptimal health behavior in the association between low education and high-risk CKM syndrome also varied by sex (Figure S4 in Multimedia Appendix 1). Regarding the educational disparity in CKM outcome, the proportion mediated by suboptimal behavior was higher for women (10.28%) than for men (8.52%), of which suboptimal physical activity and suboptimal nicotine exposure accounted for the largest proportions, respectively.

### Educational Inequalities in High-Risk CKM Syndrome in Women and Men

**Figure 4.**
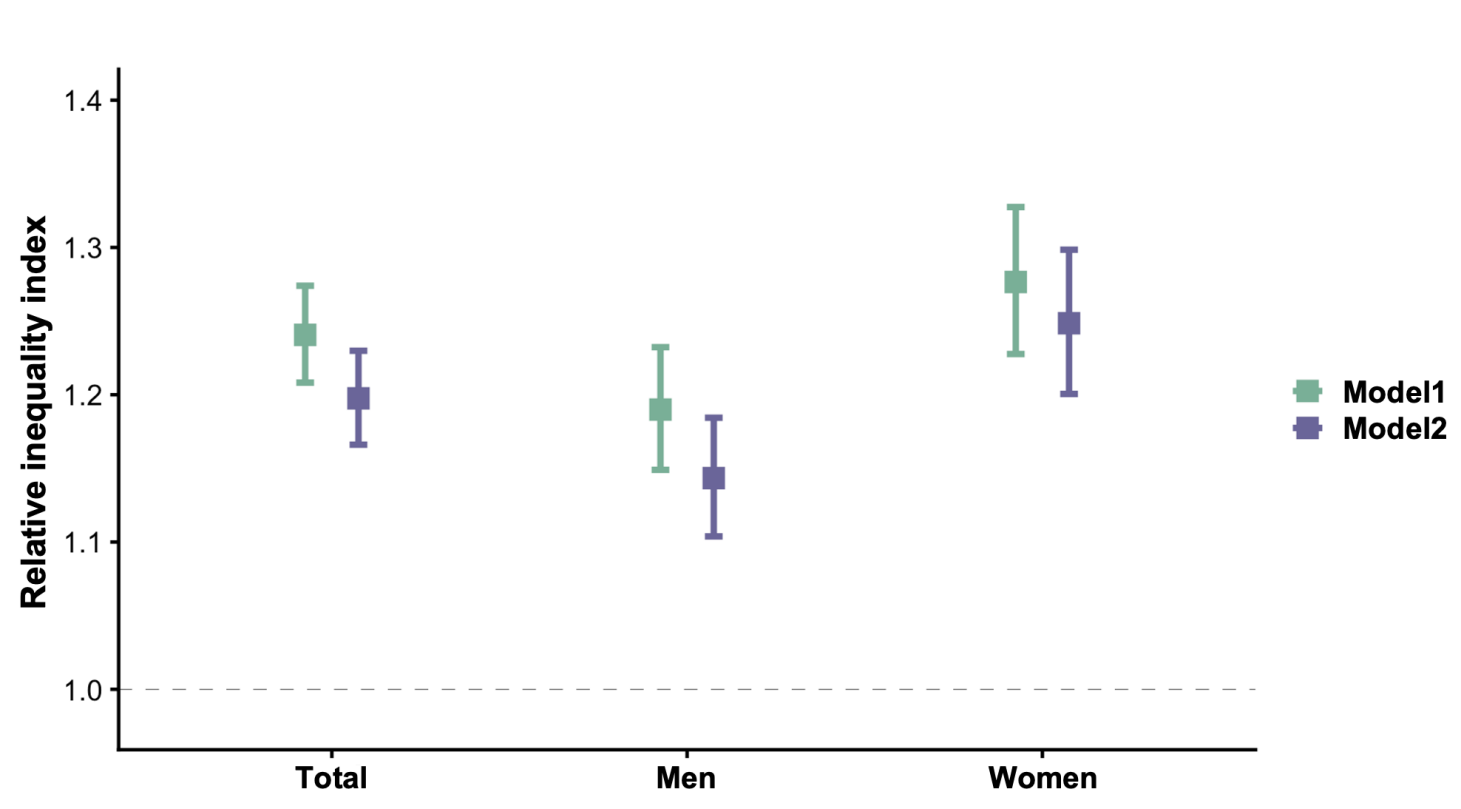
Educational relative inequality index on high-risk cardiovascular-kidney-metabolic (CKM) syndrome in the total population as well as in men and women. Analyses were conducted among participants with complete information on exposure and outcomes (n=132,085). Model 1 was adjusted for age; study center; and other socioeconomic factors, including marriage, living condition, and occupation. Model 2 was additionally adjusted for Life’s Essential 8 health behavior, including nicotine exposure, physical activity, diet, and sleep, based on model 1.

Population-level educational disparities in both sexes were further assessed by RII (Figure 4). In the total population, the educational RII for high-risk CKM was 1.24 (95% CI 1.21-1.27), with a significant disparity among women (1.28, 95% CI 1.23-1.33) and men (1.19, 95% CI 1.15-1.23). Although further adjustment for LE8 behavior factors led to modest reductions in RII, it remained significant in both sexes, with a greater educational gradient in high-risk CKM syndrome for women.

## Discussion

### Principal Results

Based on our nationwide cross-sectional study, including 132,085 middle-aged and older Chinese adults, we observed a positive correlation between low education and the whole continuum of CKM syndrome in women, but only with high-risk CKM syndrome in men. For those who are already in a high-risk CKM stage, low-educated women exhibited the poorest overall cardiometabolic condition, reflected by the achievement of optimal LE8 health factor; however, women generally had better health conditions than men. The detrimental effects of low education on high-risk CKM were more pronounced in women with suboptimal health behaviors than in men. Moreover, women bore worse educational inequalities, which were only modestly attenuated by behavior adjustments. These findings underlined the differential gender susceptibility to the CKM continuum ascribed to low education, highlighting the greater disadvantage for women; they also suggested the potential additional benefits of targeted behavior modifications for women with lower levels of education.

To our knowledge, this is the first large-scale nationwide study to evaluate holistic CKM health using a novel staging construct. The comprehensive assessment of population-based CKM spectrum provided opportunities for early implementation of targeted prevention, where it was crucial to not only improve medical care but also address SDOH [9], in which education was of vital importance. Notably, the association between educational attainment and CKM outcome largely depended on sex. According to a recent meta-analysis, women with the lowest education were at a 24% higher excess risk for coronary heart disease than men, but this increased risk was not observed for stroke [19]. As for diabetes, an inverse relation between educational attainment and diabetes risk still existed for women, but results for men varied among countries, with a weaker or insignificant association in high-income countries [28-30] and even a reversed association in low- or middle-income country [16,31,32]. Our evidence supported the synchronicity between poor education and the whole CKM spectrum in women, but an inconsistency was observed in men; specifically, women with less education were significantly more susceptible to moderate-risk CKM syndrome, while less educated men were only more likely to have high-risk CKM but not likely to have moderate-risk CKM. A possible explanation may be the disadvantaged socioeconomic status of women, including fewer social support resources and employment opportunities, as well more caregiving responsibilities and psychosocial stress [33], resulting in fewer chances to gain health awareness, develop healthy habits, and receive primary CVD prevention [34]. Thus, it is plausible that less educated women are more likely to confront excess or dysfunctional adipose and metabolic risk factors, such as metabolic syndrome [35], which are the upstream of CKM abnormalities. Although we did not demonstrate excess odds of subclinical and clinical CVD attributed to poor education in women compared to men, as reported in high-income countries [19,34], poorly educated women at an advanced stage had the lowest achievement of LE8 health factors. This re-emphasized the predominance of educational attainment in female CKM health. Our findings address the research gap in understanding sex differences concerning CKM syndrome, particularly the role of education as a crucial SDOH in CKM progression and management.

Lifestyle played an essential role in the association between education and health outcomes [13,36,37]. However, few studies investigated the sex differences in the interactive and joint association of education and lifestyle with CKM. Our study supported the female-specific harm of low education in those with poor health behaviors, which diminished with behavior improvements. This was strengthened by a stronger joint influence of behavior and education in women. Mediation analysis further confirmed that suboptimal health behavior mediated a greater proportion of the association between low education and high-risk CKM in women than in men, which was in agreement with evidence on CVD incidence or death in western countries [26,38,39]. Our findings supported the notion that sex-specific educational inequality could be partly offset by behavior improvements, especially for women. Public health should not only focus on lifestyle improvement for women already at an educational disadvantage but also on health education for women with unhealthy lifestyles, to alleviate or prevent adverse CKM outcomes. Furthermore, we identified varying contributions of each behavior component to CKM outcome between sexes, where suboptimal smoking had the strongest mediating effect in men and suboptimal physical activity had the strongest mediating effect in women. In slight contrast to previous evidence, which found that socioeconomic inequality was mostly driven by smoking in both sexes, our results supported different sex-specific targeted behavior modification strategies to aid in improving CKM health conferred by low education in Chinese participants. Finally, in line with findings from NHANES and UKBiobank [13], it should be noted that the mediation effect of an unhealthy lifestyle was relatively weak, which underscored the importance of addressing sex-specific SDOH inequalities at their source through policy interventions rather than relying solely on individual behavior improvements.

### Limitations

Several limitations should also be acknowledged. First, we only used risk equivalents of subclinical CVD to determine CKM stage 3 due to the absence of imaging markers, cardiac biomarkers, or echocardiographic parameters in our data; the proportion of stage 3 CKD could thus be underestimated. Additionally, educational level, other socioeconomic factors, LE8 health behaviors, and clinical CVD were self-reported, which could cause recall bias and reduce our statistical power. Nevertheless, face-to-face interviews and structural questionnaires were used for data collection, and CVD history was validated by an adjudication committee, which partly ensured the accuracy of self-reported data. Second, due to the cross-sectional design of the study, we could not analyze the time-varying effects of behavior factors or their interaction with incident CKM syndrome or subsequent CVD cases, so reverse causation could not be ruled out. Future prospective studies are warranted to confirm causality.

### Conclusions

In conclusion, evidence from this large-scale cohort, which is predominantly composed of women from a transitioning country with lower educational levels, shows that women with educational disadvantages have a higher odds of moderate-risk CKM syndrome than men, although this is not the case for high-risk CKM. Nevertheless, women with high-risk CKM had the poorest control of LE8 health factors. The association between low education and advanced CKM stage in women was more evident in those with poor health behaviors and was also more likely to be explained and modulated by behavior modification. Our study highlighted the prominent impact of education on CKM health in both sexes, especially in women, and the necessity to address sex-specific educational disparity. Public health considerations for CKM health improvement could not depend solely on lifestyle modifications but should also consider gender-specific differences and SDOH-related vulnerabilities.

## Supplementary material

10.2196/57920 Multimedia Appendix 1 Supplementary material.

## Abbreviations

AHA: American Heart Association
CKD: chronic kidney disease
CKM: cardiovascular-kidney-metabolic
CVD: cardiovascular disease
LE8: Life’s Essential 8
OR: odds ratio
REACTION: Risk Evaluation of Cancers in Chinese Diabetic Individuals: a Longitudinal Study
RII: relative index of inequality
ROR: ratio of odds ratio
SDOH: social determinants of health
