# Supplementary material for: Gender Disparities in the Association Between Educational Attainment and Cardiovascular-Kidney-Metabolic Syndrome: Cross-Sectional Study
Source: JMIR Public Health Surveill. 2024 Aug 23;10:e57920. doi: 10.2196/57920 (PMC11363879; doi:10.2196/57920)
Supplement: Multimedia Appendix 1 [file publichealth-v10-e57920-s001.docx]

**Multimedia Appendix 1**

Methods

**Multimedia Appendix 1 Figure S1.** Participants flowchart

**Multimedia Appendix 1 Figure S2.** Distribution of CKM syndrome stages in men and women by education level

**Multimedia Appendix 1 Figure S3.** Distribution of LE8 health behavior score in men and women by education level

**Multimedia Appendix 1 Figure S4.** Mediation analysis of the effect of LE8 health behavior on high-risk CKM syndrome (stage 3 and 4)

**Multimedia Appendix 1 Table S1.** Baseline characteristics of the study participants and participants who were excluded from the analysis

**Multimedia Appendix 1 Table S2.** Definition of CKM syndrome stages

**Multimedia Appendix 1 Table S3.** Definition of measurement and assessment of ‘Life’s Essential 8’ cardiovascular health metrics

**Multimedia Appendix 1 Table S4.** Achievement of optimal LE8 health behavior and health factor targets in men and women with different education levels, according to different CKM stages

**Multimedia Appendix 1 Table S5.** Association of low education with high-risk CKM syndrome (stage 3 and 4) in men and women with different LE8 health behavior

**Multimedia Appendix 1 Table S6**. Joint associations of education level and LE8 health behavior with high-risk CKM syndrome (stage 3 and 4) in men and women

**Multimedia Appendix 1 Table S7.** Sensitivity Analysis: Sex-specific odds ratios (and 95% CIs) and women-to-men ratio of odds ratios for moderate-risk and high-risk CKM syndrome attributable to low education (elementary school or below, middle school, high school versus college or above)

**Multimedia Appendix 1 Table S8.** Sensitivity Analysis: Association of low education with high-risk CKM syndrome (stage 3 and 4) in men and women with different LE8 health behavior (elementary school or below, middle school, high school versus college or above)

**Multimedia Appendix 1 Table S9.** Sensitivity Analysis: Joint associations of education level and LE8 health behavior with high-risk CKM syndrome (stage 3 and 4) in men and women

**Multimedia Appendix 1 Table S10.** Sensitivity Analysis: Sex-specific odds ratios (and 95% CIs) and women-to-men ratio of odds ratios for moderate-risk and high-risk CKM syndrome attributable to low education in multiple imputation data sets imputed for missing baseline information and outcome

**Multimedia Appendix 1 Table S11.** Sensitivity Analysis: Achievement of optimal LE8 health behavior and health factor targets in men and women with different education levels, according to different CKM stages in multiple imputation data sets imputed for missing baseline information and outcome

**Multimedia Appendix 1 Table S12.** Sensitivity Analysis: Association of low education with high-risk CKM syndrome (stage 3 and 4) in men and women with different LE8 health behavior in multiple imputation data sets imputed for missing baseline information and outcome

**Multimedia Appendix 1 Table S13.** Sensitivity Analysis: Joint associations of education and LE8 health behavior with high-risk CKM syndrome (stage 3 and 4) in men and women in multiple imputation data sets imputed for missing baseline information and outcome

**Multimedia Appendix 1.** Methods

**Data collection and measurements**

Baseline examinations were collected by face-to-face interviews along with a structured questionnaire, anthropometric measurements, and blood sampling. The in-person interview was conducted by trained study personnel, and the information collected included sociodemographic characteristics, behavior factors and self-reported medical history and current medications. Socio-economic factors of interest including education level (no formal education, elementary school, middle school, high school, technical school/college or above), marital status (married/cohabitant, never married, widowed, separated/divorced, others, unknown), living status (with spouse and children, with spouse only, with children only, with others, alone) and occupation (manufacturing, agricultural, professional/technical/administrative, clerical/commercial/service, unemployed/household, retired, others, unknown) were recorded.

**Measurements**

For enrolled participants, the clinic visit took place at the local health stations or community clinics in the participants’ residential area. All physical examinations were scheduled in the morning. Anthropometric measurements were taken with participants wearing light clothing and no shoes. Height was measured using a calibrated stadiometre with subjects standing fully upright with heels together. Weight was measured using a calibrated scale that was carefully zeroed before each measurement. Body mass index (BMI) was calculated as weight in kilogrammes divided by height in metres squared (kg/m^2^).

An automated electronic device (OMRON Model HEM-752 FUZZY; Omron Co) was used to measure systolic blood pressure (SBP) and diastolic blood pressure (DBP) three times consecutively at 1-min intervals after a rest of at least 5 min, and the average of three readings on the same day was used in the analysis.

All participants were asked to fast overnight for ≥ 10 h before blood samples were taken. A standard Oral glucose tolerance test (OGTT) was conducted, and fasting and 2-hour post-load plasma glucose (2h-PG) concentrations were measured at the relevant local hospitals. Serum samples were aliquoted into 0.5-mL Eppendorf tubes within 2h and shipped by air in dry ice to the central laboratory located at the Shanghai Institute of Endocrine and Metabolic Diseases, which was certified by the College of American Pathologists (CAP). The level of haemoglobin A1c (HbA1c) was determined by the method of high-performance liquid chromatography (VARIANT II and D-10Systems; Bio-Rad).

Lipid profiles. including total cholesterol (TC), low-density lipoprotein cholesterol (LDL-c), high-density lipoprotein cholesterol (HDL-c) and triglycerides (TGs), were measured with an autoanalyser (Abbott Laboratories, IL, USA).

Urinary albumin (immunonephelometry using Siemens BNII and BN ProSpec nephelometers [Siemens Healthcare Diagnostics, Marburg, Germany]) and urinary creatinine concentrations were tested (by enzymatic method [ADVIA Chemistry XPT System; Siemens Healthcare, Erlangen, Germany]) for calculation of Urinary albumin-to-creatinine ratio (ACR). Serum creatinine was also used to calculate estimated glomerular filtration rate (GFR) based on the Chronic Kidney Disease Epidemiology Collaboration (CKD-EPI) equation for Asian individuals.

**Statistical analysis**

Baseline characteristics of participants were summarized by sex and presented as means (standard deviation) or medians (interquartile ranges) for continuous measures, and numbers (proportions, %) for categorical measures. Distributions of CKM syndrome stages in different educational subgroups were visualized by stacked bar plot in men and women separately.

We conducted stratified analyses to investigate associations of education level with high-risk CKM syndrome (stage 3 and 4) among men and women in different Life’s Essential 8 (LE8) health behavior groups (0-1 optimal health behavior, 2 optimal health behaviors and 3-4 optimal health behaviors). In each group, ORs of high-risk CKM attributed to low education were calculated with high education as a reference. We additionally included a product term of education level and health behavior group to quantify the additive and multiplicative interactions^1^ between the health behavior groups (0-1 optimal health behavior versus 3-4 optimal health behaviors) and education (low versus high), which were odds ratio of the product term and relative excess risk due to interaction (RERI), respectively. The multiplicative interaction was statistically significant when its confidential interval (CI) did not include 1, while additive interaction was statistically significant when its CI did not include 0.

Joint associations of the combination of education and LE8 health behavior in men and women were also evaluated. Participants were categorized into six groups according to education (low and high) and health behavior groups (0-1, 2 and 3-4), with the combination of high education and 3-4 optimal health behaviors as reference. Odds ratios of high-risk CKM syndrome in different groups were calculated. The interaction term between the combined education-behavior group and sex was also added to obtain P for interaction and women-to-men ratio of odds ratio (ROR).

The potential mediating effects of each suboptimal behavior defined LE8 were analyzed by multiple parallel mediation model in men and women, respectively, utilizing R package *bruceR* ^2^. All suboptimal health behaviors (suboptimal nicotine exposure, suboptimal diet, suboptimal physical activity, and suboptimal sleep) were included in the model. The mediating effect of the overall suboptimal health behavior score (sum of 4 suboptimal health behaviors, ranging from 0 to 4 points) was also calculated. We used the bootstrapping procedure to measure the indirect effect and estimated 95% CIs based on 1000 bootstrap samples. The proportion mediated was calculated by dividing the indirect effect by total effect.

Relative index of inequality (RII) was utilized to quantify the educational gradient in relative terms. We measured the relative educational rank of each individual by proportion of our population that has a higher educational position. In the binomial regression model with this transformed variable as an exposure and high-risk CKM syndrome as outcome, RII was calculated using the natural logarithm link function, and CIs were constructed by robust SE estimator.

Missing values were handled in the following approaches: (1) Since the purpose of our study was mainly to explore the association of education level and CKM syndrome, we included 132085 participants with complete information on education and CKM syndrome stages in main analyses, where all covariates with missing values, including socioeconomic factors (marital status, living status and occupation) with a missing proportion of less than 10%, LE8 health behaviors, and medication information, were recoded the as another category (unknown); (2) As for subgroup analyses concerning LE8 metrics, we excluded those with any missing in LE8 health behavior factors, resulting in a complete-case analyzing sample of N=99124. (3) Finally, considering the relatively large proportion of exclusion due to missing data, we used multiple imputation to impute for missing values on all exposures, outcomes and covariates to validate the robustness of our results in the sensitivity analyses.

1. Zhang YB, Chen C, Pan XF, et al. Associations of healthy lifestyle and socioeconomic status with mortality and incident cardiovascular disease: two prospective cohort studies. BMJ. Apr 14 2021;373:n604. doi:10.1136/bmj.n604

2. Bao, H.-W.-S. bruceR: Broadly useful convenient and efficient R functions (Version 2023.9). Sep 2023; https://CRAN.R-project.org/package=bruceR

**Multimedia Appendix 1 Figure S1. Participant flowchart** **
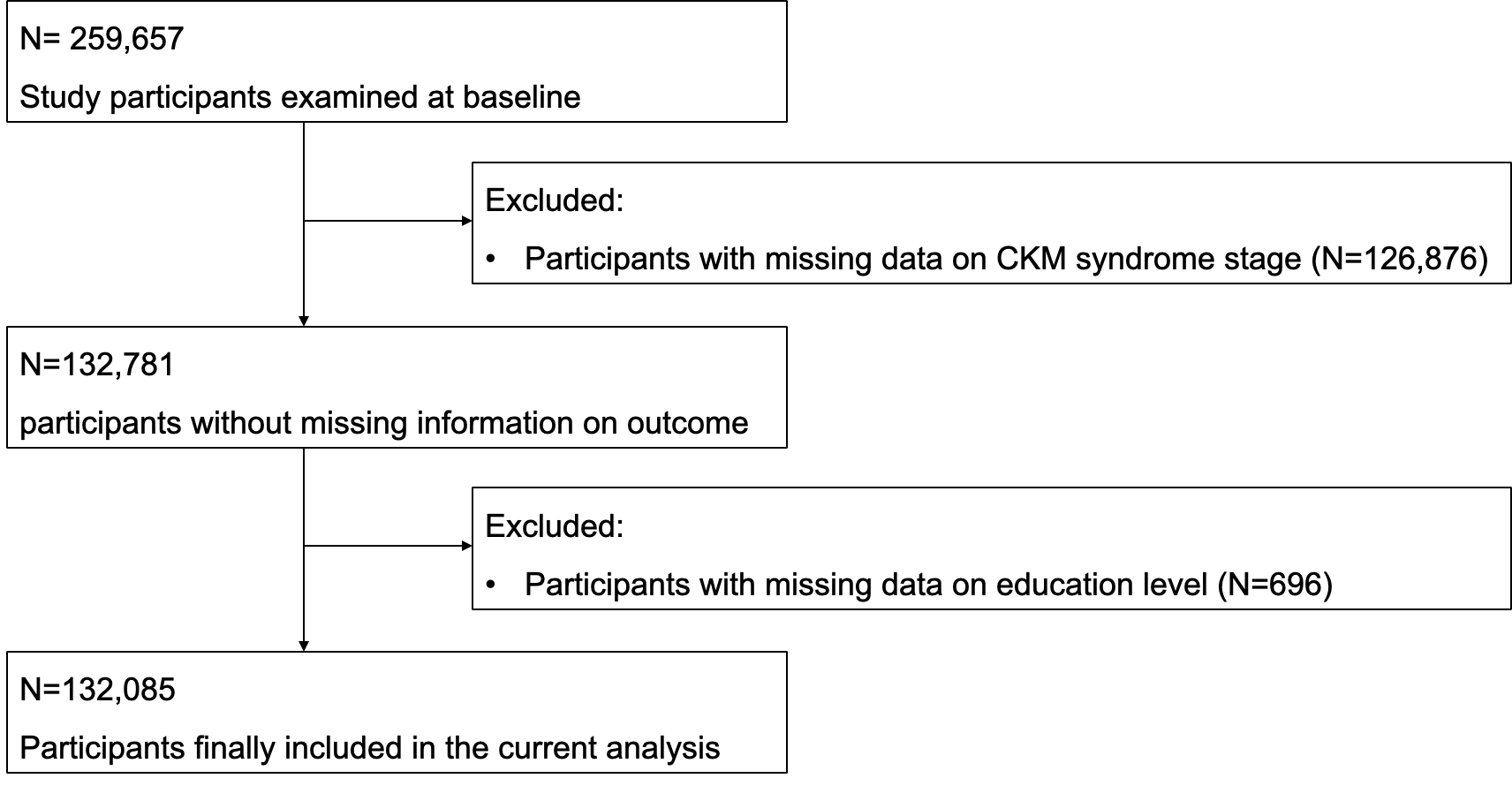
**

**Multimedia Appendix 1 Figure S2. Distribution of CKM** **syndrome stages in men and women by education level**

**
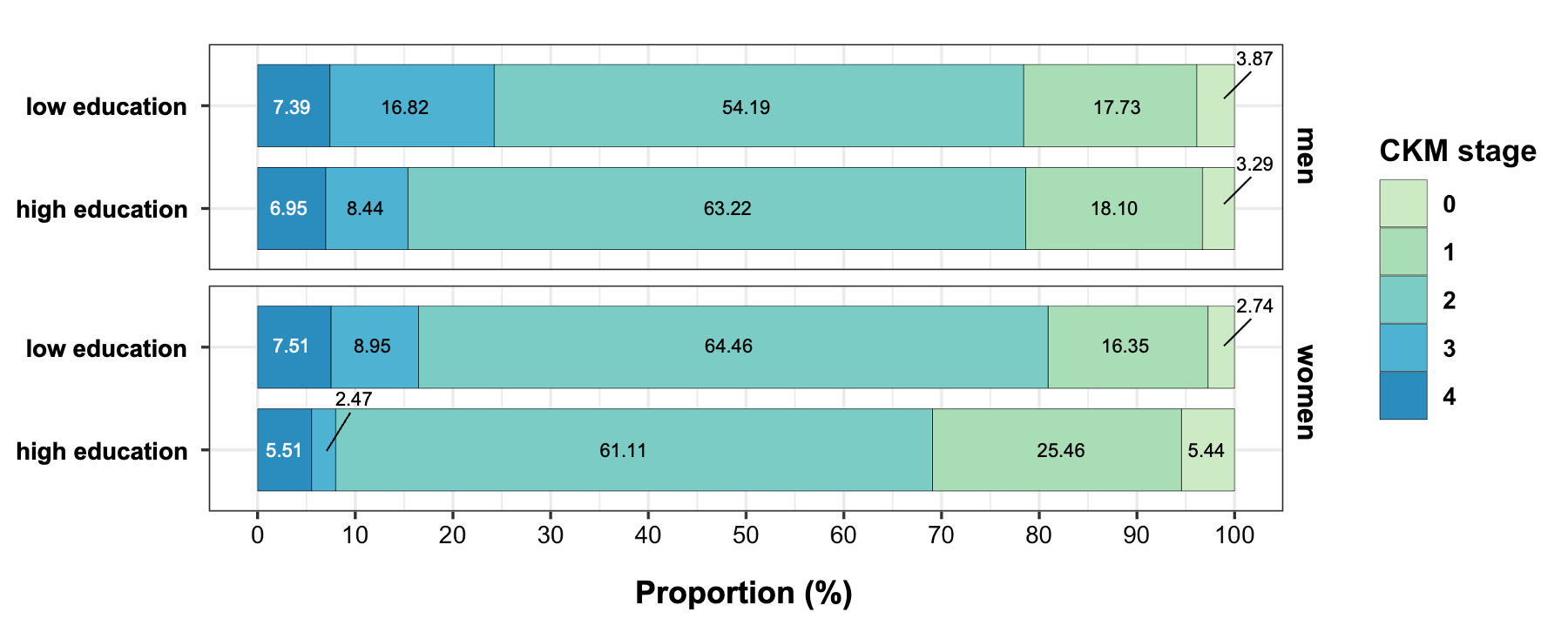
**

CKM, cardiovascular-kidney-metabolic.

**Multimedia Appendix 1 Figure S3. Distribution of LE8 health behavior score in men and women by education level**

**
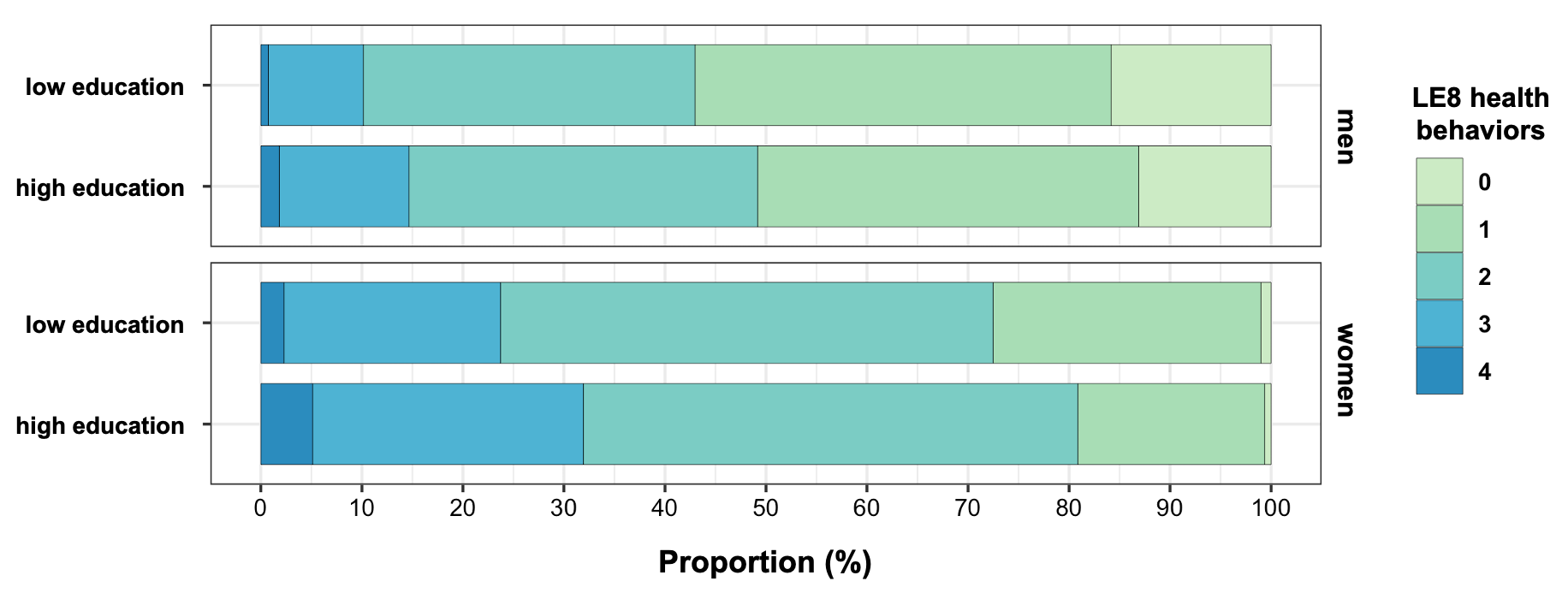
**

LE8, Life’s Essential 8.

**Multimedia Appendix 1 Figure S4. Mediation analysis of the effect of LE8 health behavior on high-risk CKM syndrome (stage 3 and 4)**

LE8, Life’s Essential 8. CKM, cardiovascular-kidney-metabolic.

Models were adjusted for age, study center, other socioeconomic factors including marital status, living status and occupation, as well as medication information including hypoglycemic drugs, antihypertensive drugs and lipid-lowering drugs (use or not) in men and women, respectively.

**Multimedia Appendix 1 Table S1. Baseline characteristics of the study participants and participants who were excluded from the analysis**

| **Characteristic** | **Study Participants ^a^** | **Excluded 1 ^b^** | **Excluded 2 ^c^** |
| --- | --- | --- | --- |
| Number of participants | 132085 | 126876 | 696 |
| Age, years | 56.9 ± 9.1 | 57.9 ± 10.0 | 57.1 ± 9.2 |
| Low education, n (%) | 40,234 (30.5) | 43,961 (33.1) | / |
| Men, n (%) | 45,410 (34.4) | 46,235 (34.8) | 214 (34.2) |
| Body-mass index, kg/m2 | 24.6 ± 3.6 | 24.7 ± 3.6 | 25.2 ± 3.8 |
| Waist circumference, cm | 84.4 ± 9.9 | 84.3 ± 9.9 | 85.0 ± 10.3 |
| Systolic BP, mmHg | 133.3 ± 21.0 | 133.1 ± 20.5 | 135.8 ± 21.1 |
| Diastolic BP, mmHg | 78.6 ± 11.2 | 77.7 ± 11.2 | 78.9 ± 11.7 |
| Fasting glucose, mg/dl | 107.3 ± 29.6 | 106.9 ± 29.7 | 107.6 ± 28.5 |
| Post-load glucose, mg/dl | 149.3 ± 69.5 | 149.7 ± 69.2 | 149.0 ± 65.2 |
| HbA1c, % | 6.0 ± 1.0 | 6.0 ± 1.0 | 6.1 ± 1.0 |
| Total cholesterol, mg/dl | 191.4 ± 44.4 | 190.8 ± 42.5 | 200.6 ± 42.5 |
| LDL cholesterol, mg/dl | 110.6 ± 34.0 | 111.1 ± 32.9 | 118.5 ± 34.0 |
| HDL cholesterol, mg/dl | 51.0 ± 13.7 | 52.2 ± 14.1 | 52.3 ± 13.7 |
| Triglycerides, mg/dl | 117.8 (83.3-171.9) | 112.5 (80.6-163.0) | 122.7 (86.8-178.1) |

Values are mean (SD) for continuous variables and number (proportion) for categorical variables.

^a^ Study participants included in the current analyses (N = 132085; Please refer to the Multimedia Appendix 1 Figure 1 for a flowchart of the study participants).

^b^ Participants who were excluded due to missing data on CKM stage (N = 126876; Please refer to the Multimedia Appendix 1 Figure 1 for a flowchart of the study participants)

^c^ Participants who were excluded due to missing data on education level (N = 696; Please refer to the Multimedia Appendix 1 Figure 1 for a flowchart of the study participants)

**Multimedia Appendix 1 Table S2. Definitions of CKM Syndrome Stages**

| **CKM Syndrome Stages** | **Definition** |
| --- | --- |
| Stage 0: No CKM risk factors | Individuals with normal BMI and waist circumference, normoglycemia, normotension, a normal lipid profile, and no evidence of CKD or subclinical or clinical CVD |
| Stage 1: Excess or dysfunctional adiposity | Individuals with overweight/obesity, abdominal obesity, or dysfunctional adipose tissue, without the presence of other metabolic risk factors or CKD ^e^ (moderate- or high- risk)   - BMI ≥23 kg/m^2^ - Waist circumference ≥80/90 cm in women/ men, or - Prediabetes^a^ |
| Stage 2: Metabolic risk factors and CKD | Individuals with metabolic risk factors:   - Diabetes^a^ - Hypertension^b^, - Metabolic Syndrome^c^, - Dyslipidemia^d^,   or CKD ^e^ (moderate- or high-risk) |
| Stage 3: Subclinical CVD^f^ in CKM | Risk equivalents of subclinical CVD   - Very high-risk CKD ^e^ - High predicted 10-y CVD risk (≥20%) ^g^ |
| Stage 4: Clinical CVD in CKM | Clinical CVD (coronary heart disease, myocardial infarction, stroke or peripheral artery disease) |

CKM, cardiovascular-kidney-metabolic; BMI, body mass index; CKD, chronic kidney disease; CVD, cardiovascular disease.

^a^ Prediabetes and diabetes are defined according to the 2020 American Diabetes Association (ADA) criteria. Specifically, prediabetes is diagnosed if fasting plasma glucose 5.6–6.9 mmol/L, or 2-hour post-load plasma glucose (2h-PG) 7.8 to 11.0 mmol or hemoglobin A1c (HbA1c) 5.7% to 6.4%; diabetes is diagnosed if fasting plasma glucose ≥ 7 mmol/L, or 2h-PG≥ 11 mmol/L, or HbA1c ≥ 6.5%, or a prior diagnosis by physicians; other participants were considered to have normal glucose regulation (NGR).

^b^ Hypertension is defined as systolic blood pressure≥130 mmHg or diastolic blood pressure ≥ 80 mmHg or under anti-hypertensive medication.

^c^ Metabolic Syndrome is defined by the presence of 3 or more of the following: (1) waist circumference≥80 cm for women and ≥90 cm for men; (2) high-density lipoprotein (HDL) cholesterol <40 mg/dL for men and <50 mg/dL for women; (3) triglycerides ≥150 mg/dL; (4) elevated blood pressure (systolic blood pressure ≥130 mmHg or diastolic blood pressure ≥80 mmHg and/or use of antihypertensive medications); and (5) fasting blood glucose ≥100 mg/dL.

^d^ Dyslipidemia is defined as total cholesterol≥ 240 mg/dL or low-density lipoprotein (LDL) cholesterol ≥ 160 mg/dL or HDL cholesterol < 40 mg/dL or under lipid-lowering medication.

^e^ CKD classification is defined according to Kidney Disease Improving Global Outcomes (KDIGO) 2012 recommendations. GFR contains a 5-stage classification (G1 to G5), while albumin-to-creatinine ratio (ACR) was classified into 3 categories (A1 to A3) as shown below. Participants with G1–2 and A1 were defined at low risk for CKD; Participants with G1–2 and A2, or G3a and A1 were defined at moderate risk for CKD; Participants with G1–2 and A3, or G3a and A2, or G3b and A1 were defined at high risk for CKD; Participants with G4–5 and A1, or G3b–5 and A2, or G3a–5 and A3 were defined at very high risk for CKD.


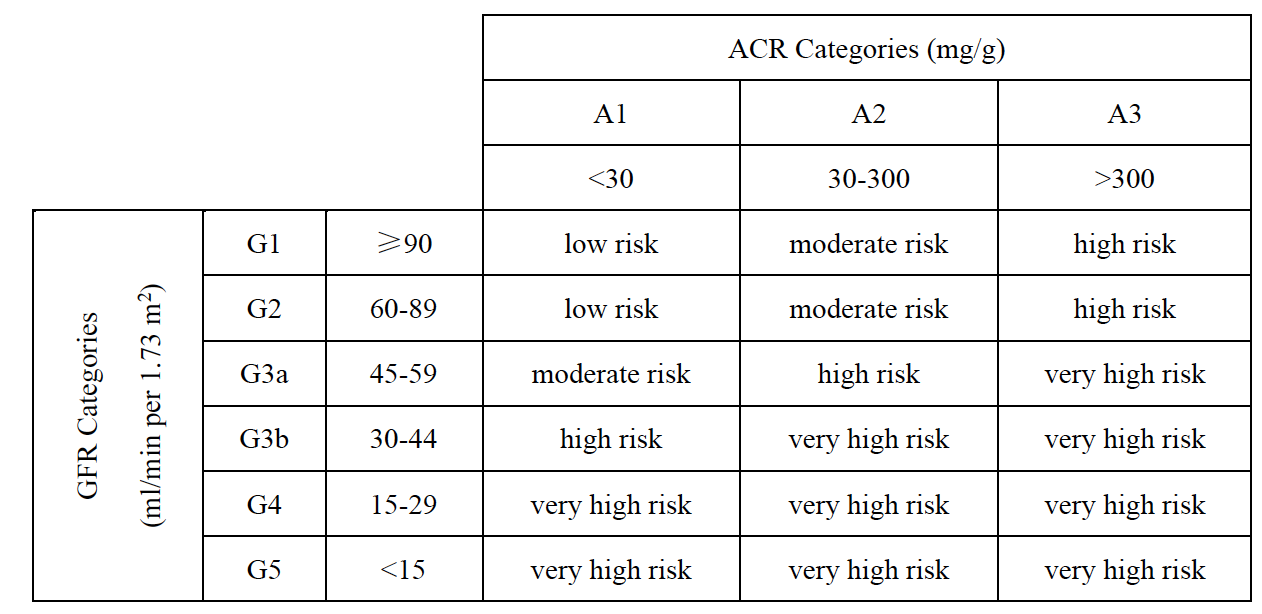


^f^ Given measurements to diagnose subclinical atherosclerotic cardiovascular disease (ASCVD) or heart failure are not available in our cohort, we use risk equivalents of subclinical CVD instead.

^g^ Predicted 10-year total CVD risk is calculated by Development and Validation of the American Heart Association Predicting Risk of Cardiovascular Disease EVENTs (PREVENT) Equations by *Khan et al.* We used the additional equation which include optional predictors (urine ACR and HbA1c).

**Multimedia Appendix 1 Table S3. Definition of measurement and assessment of ‘Life’s Essential 8’ cardiovascular health metrics**

| CVH metric | Method of measurement | Quantification of CVH metrics |
| --- | --- | --- |
| Nicotine exposure | Self-reported cigarettes smoking | Points Tobacco use of secondhand smoke exposure  100: Never smoker  75: Former smoker, quit ≥5 years  50: Former smoker, quit 1–<5 years  25: Former smoker, quit <1 years  0: Current smoker  Subtract 20 points (unless score is 0) for living with active indoor smoker in home |
| Physical activity | Self-reported physical activity | Points Minutes of moderate or vigorous physical activity per week  100: ≥150  90: 120–149  80: 90–119  60: 60–89  40: 30–59  20: 1–29  0: 0 |
| Diet | Self-reported healthy diet of 7 components^a^ | Points Number of healthy diet components  100: 6 or 7  80: 5 or 4  50: 3  25: 2  0: 0 or 1 |
| Sleep  health | Self-reported average hours of sleep per day | Points Hours of sleep per day  100: 7-<9  90: 9-<10  70: 6-<7  40: 5-<6 or ≥10  20: 4–<5  0: <4 |
| BMI | Objective measurement of weight and height | Points BMI (kg/m^2^)  100: <23.0  75: 23.0-<25.0  50: 25.0-<30.0  25: 30.0-<35.0  0: ≥35.0 |
| Blood lipids | Fasting plasma non–HDL cholesterol tested at the central laboratory | Points Non-HDL cholesterol (mg/dl)  100: <130  60: 130-159  40: 160-189  20: 190-219  0: ≥220  Subtract 20 points if treated level |
| Blood glucose | Fasting plasma glucose, 2h post-load plasma glucose tested locally, and Fasting HbA1c tested at the central laboratory | Points Glycemic status  100: NGR: No history of diabetes and FPG<100 mg/dl and OGTT-2h PPG<140mg/dl and HbA1c<5.7%  60: Prediabetes: No diabetes and (FPG 100-125mg/dl or OGTT-2h PPG 140-199mg/dl or HbA1c 5.7-6.4%)  40: Diabetes with HbA1c<7.0%  30: Diabetes with HbA1c: 7.0-7.9%  20: Diabetes with HbA1c: 8.0-8.9%  10: Diabetes with HbA1c: 9.0-9.9%  0: Diabetes with HbA1c: ≥10.0% |
| Blood pressures | Objective measurement of SBP and DBP | Points SBP and DBP (mmHg)  100: <120/80 (optimal)  75: 120-129/<80 (elevated)  50: 130-139 or 80-89 (stage 1 hypertension)  25: 140-159 or 90-99  0: ≥160 or ≥100  Subtract 20 points if treated level |

CVH, cardiovascular health; BMI, body mass index; HDL, high-density lipoprotein; NGR, normal glucose regulation; FPG, fasting plasma glucose; OGTT, oral glucose tolerant test; PPG, post-load plasma glucose; HbA1c, glycated hemoglobin; SBP, systolic blood pressure; DBP, diastolic blood pressure.

^a^ The healthy diet score included the following 7 components: fruits and vegetables ≥4.5 cups/day, fish ≥two 3.5-oz servings/week, livestock and poultry meat: 40-75g/day, sweets/sugar-sweetened beverages ≤450 kcal/week, soy protein ≥25 g/day, milk or yogurt ≥3 cups/week, alcohol ≤ 2 servings per day for men or ≤ 1 serving per day for women.

**Multimedia Appendix 1 Table S4. Achievement of optimal LE8 health behavior and health factor targets in men and women with different education levels, according to different CKM stages**

|  | **No/Total (%)** | **Optimal health behavior** | **No/Total (%)** | **Optimal health factors** |
| --- | --- | --- | --- | --- |
| **All participants** |  |  |  |  |
| Men, high education | 2391/25856 (9.2) | 9.90 (9.5, 10.32) | 5584/34564 (16.2) | 12.36 (12.00, 12.73) |
| Men, low education | 444/7138 (6.2) | 6.78 (6.19, 7.43) | 1852/10714 (17.3) | 17.67 (16.9, 18.47) |
| Women, high education | 7600/45683 (16.6) | 17.49 (17.07, 17.92) | 14211/57097 (24.9) | 18.18 (17.82, 18.56) |
| Women, low education | 2651/20447 (13.0) | 14.03 (13.49, 14.59) | 4367/29418 (14.8) | 14.28 (13.84, 14.73) |
| **CKM syndrome Stage 0** |  |  |  |  |
| Men, high education | 88/828 (10.6) | 10.83 (8.7, 13.39) | 1001/1140 (87.8) | 88.01 (85.65, 90.02) |
| Men, low education | 24/294 (8.2) | 8.06 (5.34, 12.01) | 361/416 (86.8) | 88.96 (85.35, 91.76) |
| Women, high education | 431/2384 (18.1) | 18.95 (17.00, 21.07) | 2851/3113 (91.6) | 90.29 (88.87, 91.55) |
| Women, low education | 88/528 (16.7) | 17.02 (13.72, 20.92) | 720/807 (89.2) | 89.84 (87.24, 91.95) |
| **CKM syndrome Stage 1** |  |  |  |  |
| Men, high education | 496/4800 (10.3) | 11.34 (10.37, 12.40) | 2574/6276 (41.0) | 38.43 (37.09, 39.78) |
| Men, low education | 96/1397 (6.9) | 7.58 (6.22, 9.22) | 816/1904 (42.9) | 43.64 (41.27, 46.04) |
| Women, high education | 2184/11781 (18.5) | 19.95 (19.06, 20.86) | 7176/14556 (49.3) | 44.67 (43.71, 45.64) |
| Women, low education | 532/3620 (14.7) | 16.08 (14.75, 17.49) | 2093/4823 (43.4) | 42.07 (40.52, 43.64) |
| **CKM syndrome Stage 2** |  |  |  |  |
| Men, high education | 1524/16337 (9.3) | 9.69 (9.19, 10.20) | 1765/21837 (8.1) | 5.94 (5.63, 6.27) |
| Men, low education | 255/3947 (6.5) | 6.62 (5.87, 7.46) | 478/5803 (8.2) | 7.51 (6.86, 8.22) |
| Women, high education | 4575/27997 (16.3) | 16.88 (16.36, 17.42) | 3912/34873 (11.2) | 8.51 (8.19, 8.85) |
| Women, low education | 1801/13180 (13.7) | 14.02 (13.36, 14.70) | 1382/18950 (7.3) | 6.85 (6.47, 7.25) |
| **CKM syndrome Stage 3** |  |  |  |  |
| Men, high education | 155/2221 (7.0) | 7.71 (6.52, 9.10) | 89/2913 (3.1) | 1.58 (1.19, 2.10) |
| Men, low education | 49/1076 (4.6) | 5.00 (3.75, 6.64) | 116/1799 (6.4) | 2.45 (1.84, 3.24) |
| Women, high education | 140/1197 (11.7) | 12.86 (10.86, 15.15) | 35/1411 (2.5) | 1.45 (0.99, 2.12) |
| Women, low education | 124/1774 (7.0) | 7.53 (6.22, 9.08) | 69/2631 (2.6) | 0.91 (0.65, 1.26) |
| **CKM syndrome Stage 4** |  |  |  |  |
| Men, high education | 128/1670 (7.7) | 8.55 (7.12, 10.25) | 155/2398 (6.5) | 5.29 (4.43, 6.3) |
| Men, low education | 20/424 (4.7) | 5.27 (3.40, 8.08) | 81/792 (10.2) | 9.71 (7.76, 12.1) |
| Women, high education | 270/2324 (11.6) | 12.82 (11.27, 14.56) | 237/3144 (7.5) | 6.00 (5.17, 6.95) |
| Women, low education | 106/1345 (7.9) | 8.70 (7.13, 10.58) | 103/2207 (4.7) | 4.26 (3.48, 5.22) |

CKM, cardiovascular-kidney-metabolic.

Analyses were conducted in participants with complete information on LE8 health behavior (n=99124) or LE8 health factor (n=131793), respectively. Age-adjusted proportions (and 95% confidential intervals) were obtained from logistic regressions. A restricted cubic spline function was applied to age with 4 knots placed at the 5^th^, 35^th^, 65^th^, and 95^th^ percentiles.

**Multimedia Appendix 1 Table S5. Association of low education with high-risk CKM syndrome (stage 3 and 4) in men and women with different LE8 health behavior**

|  | **Men** | | **Women** | | **Women-to-men**  **ROR** | **P _for interaction_** |
| --- | --- | --- | --- | --- | --- | --- |
|  | **Cases/Total (%)** | **OR (95% CI)** | **Cases/Total (%)** | **OR (95% CI)** |  |  |
| 0-1 optimal health behavior | | | | | | |
| High education | 1838 (16.2) | 1.00 (ref.) | 586 (10.4) | 1.00 (ref.) | 1.00 (ref.) | / |
| Low education | 815 (22.8) | **1.23 (1.09, 1.39)** | 737 (22.2) | **1.63 (1.38, 1.92)** | **1.21 (1.01, 1.44)** | **0.038** |
| 2 optimal health behaviors | | | | | | |
| High education | 1461 (14.8) | 1.00 (ref.) | 1873 (8.2) | 1.00 (ref.) | 1.00 (ref.) | / |
| Low education | 519 (19.4) | **1.33 (1.15, 1.54)** | 1729 (15.3) | **1.37 (1.25, 1.51)** | 1.04 (0.90, 1.21) | 0.587 |
| 3-4 optimal health behaviors | | | | | | |
| High education | 592 (12.6) | 1.00 (ref.) | 1062 (6.2) | 1.00 (ref.) | 1.00 (ref.) | / |
| Low education | 166 (18.6) | **1.75 (1.36, 2.25)** | 653 (11.2) | **1.32 (1.15, 1.52)** | 0.84 (0.66, 1.08) | 0.164 |

CKM, cardiovascular-kidney-metabolic; LE8, Life’s Essential 8; OR, odds ratio; CI, confidential interval; ROR, ratio of odds ratio.

Analyses were conducted in participants with complete information on LE8 health behaviors (N=99124). Models were adjusted for age, study center, other socioeconomic factors including marital status, living status and occupation, as well as medication information including hypoglycemic drugs, antihypertensive drugs and lipid-lowering drugs (use or not). In each health behavior subgroup, those with high education (middle school or above) were selected as the control group. In the total population, the interaction term between education and sex was added to obtain P for interaction and the women-to-men ratio of odds ratio (ROR). Please refer to the Figure 2A for multiplicative and addictive interactive associations between LE8 health behavior and education level.

**Multimedia Appendix 1 Table S6. Joint associations of education level and LE8 health behavior with high-risk CKM syndrome (stage 3 and 4) in men and women**

|  | **Men** | | **Women** | | **P _for interaction_** |
| --- | --- | --- | --- | --- | --- |
|  | **Cases/Total (%)** | **OR (95% CI)** | **Cases/Total (%)** | **OR (95% CI)** |  |
| High education, 3-4 optimal health behaviors | 592 (12.6) | 1.00 (ref.) | 1062 (6.2) | 1.00 (ref.) | / |
| High education, 2 optimal health behaviors | 1461 (14.8) | 1.16 (1.04, 1.30) | 1873 (8.2) | 1.12 (1.03, 1.22) | 0.598 |
| High education, 0-1 optimal health behavior | 1838 (16.2) | 1.39 (1.24, 1.55) | 586 (10.4) | 1.29 (1.14, 1.45) | 0.756 |
| Low education, 3-4 optimal health behaviors | 166 (18.6) | 1.52 (1.23, 1.88) | 653 (11.2) | 1.49 (1.33, 1.68) | 0.912 |
| Low education, 2 optimal health behaviors | 519 (19.4) | 1.67 (1.43, 1.93) | 1729 (15.3) | 1.86 (1.68, 2.05) | 0.063 |
| Low education, 0-1 optimal health behavior | 815 (22.8) | 2.04 (1.78, 2.35) | 737 (22.2) | 2.58 (2.28, 2.91) | 0.001 |

CKM, cardiovascular-kidney-metabolic; LE8, Life’s Essential 8; OR, odds ratio; CI, confidential interval; ROR, ratio of odds ratio.

Analyses were conducted in participants with complete information on LE8 health behaviors (N=99124). Models were adjusted for age, study center, and other socioeconomic factors including marital status, living status and occupation, as well as medication information including hypoglycemic drugs, antihypertensive drugs and lipid-lowering drugs (use or not). The combination of high education (middle school or above) and 3-4 optimal health behaviors was selected as the control group. In the total population, the P for interaction was obtained by the product term of combined education-behavior group (low education & 0-1 optimal health behavior versus high education & 3-4 optimal health behaviors) and sex. Please refer to the Figure 2B.

**Multimedia Appendix 1 Table S7. Sensitivity Analysis: Sex-specific odds ratios (and 95% CIs) and women-to-men ratio of odds ratios for moderate-risk and high-risk CKM syndrome attributable to low education (elementary school or below, middle school, high school versus college or above)**

|  | **Cases/Total**  **(%)** | **MODEL 1** | | | | **MODEL 2** | | | |
| --- | --- | --- | --- | --- | --- | --- | --- | --- | --- |
|  |  | **Men**  **OR (95% CI)** | **Women**  **OR (95% CI)** | **Women-to-men ROR** | **P _for interaction_** | **Men**  **OR (95% CI)** | **Women**  **OR (95% CI)** | **Women-to-men ROR** | **P _for interaction_** |
| OR for the prevalence of moderate-risk CKM syndrome | | | | | | | | | |
| College or above | 10472 (81.4) | 1.00 (ref.) | 1.00 (ref.) | / | / | 1.00 (ref.) | 1.00 (ref.) | / | / |
| High school | 28473 (85.0) | 0.84 (0.69, 1.03) | **1.17 (1.03, 1.32)** | **1.50 (1.21, 1.87)** | **<0.0001** | 0.87 (0.71, 1.06) | **1.17 (1.03, 1.33)** | **1.46 (1.17, 1.82)** | 0.0008 |
| Middle school | 38749 (85.2) | 0.88 (0.72, 1.08) | **1.65 (1.45, 1.88)** | **2.21 (1.79, 2.73)** | **<0.0001** | 0.93 (0.76, 1.15) | **1.66 (1.45, 1.9)** | **2.09 (1.69, 2.59)** | **<0.0001** |
| Elementary school or below | 31558 (78.4) | 0.78 (0.62, 0.97) | **1.95 (1.67, 2.27)** | **4.28 (3.43, 5.36)** | **<0.0001** | 0.83 (0.66, 1.04) | **1.96 (1.68, 2.28)** | **3.92 (3.14, 4.92)** | **<0.0001** |
| OR for the prevalence of high-risk CKM syndrome | | | | | | | | | |
| College or above | 1753 (13.6) | 1.00 (ref.) | 1.00 (ref.) | / | / | 1.00 (ref.) | 1.00 (ref.) | / | / |
| High school | 3284 (9.8) | 1.01 (0.91, 1.11) | 1.03 (0.92, 1.16) | 1.03 (0.89, 1.2) | 0.6633 | 1.06 (0.95, 1.18) | 1.07 (0.95, 1.21) | 1.05 (0.90, 1.23) | 0.511 |
| Middle school | 4867 (10.7) | 1.10 (0.999, 1.21) | 1.10 (0.98, 1.23) | 1.02 (0.89, 1.18) | 0.7692 | **1.19 (1.07, 1.32)** | **1.15 (1.02, 1.30)** | 1.02 (0.88, 1.19) | 0.784 |
| Elementary school or below | 7453 (18.5) | **1.26 (1.13, 1.40)** | **1.22 (1.09, 1.37)** | 0.98 (0.85, 1.13) | 0.7694 | **1.44 (1.28, 1.61)** | **1.28 (1.13, 1.45)** | 0.89 (0.77, 1.04) | 0.135 |

CKM, cardiovascular-kidney-metabolic; OR, odds ratio; CI, confidential interval; ROR, ratio of odds ratio.

Analyses were conducted in participants with complete information on exposure and outcome (N=132085). Model 1 was adjusted for age, study center and other socioeconomic factors including marital status, living status and occupation. Model 2 was further adjusted for Life’s Essential 8 health behavior including nicotine exposure, physical activity, diet and sleep, as well as medication information including hypoglycemic drugs, antihypertensive drugs and lipid-lowering drugs (use or not). For high-risk CKM syndrome, CKM stages 0 to 2 were used as reference; for moderate-risk CKM syndrome, CKM stages 0 was used as reference. Odds ratio was calculated by contrasting lower education level (high school, middle school, elementary school or below) with the highest education level (college or above), in men and women, respectively. In total population, the interaction term between education and sex was added to obtain P for interaction and the women-to-men ratio of odds ratio (ROR).

**Multimedia Appendix 1 Table S8.** **Sensitivity Analysis: Association of low education with high-risk CKM syndrome (stage 3 and 4) in men and women with different LE8 health behavior (elementary school or below, middle school, high school versus college or above)**

|  | **Men** | | **Women** | | **Women-to-men**  **ROR** | **P _for interaction_** |
| --- | --- | --- | --- | --- | --- | --- |
|  | **Cases/Total (%)** | **OR (95% CI)** | **Cases/Total (%)** | **OR (95% CI)** |  |  |
| Multiplicative and additive interaction between health behavior and education level | OR for product term 1.17  95% CI 0.87 to 1.58.  RERI 0.30, 95% CI -0.06 to 0.65 | | OR for product term 1.48,  95% CI 1.03 to 2.13.  RERI 0.74, 95% CI 0.33 to 1.14 | | **/** | **/** |
| 0-1 optimal health behavior | | | | | | |
| College or above | 350 (17.5) | 1.00 (ref.) | 68 (11.3) | 1.00 (ref.) | 1.00 (ref.) | / |
| High school | 596 (16.0) | 1.09 (0.92, 1.29) | 211 (9.8) | 1.01 (0.73, 1.41) | 0.91 (0.64, 1.31) | 0.617 |
| Middle school | 892 (15.9) | 1.07 (0.91, 1.26) | 307 (10.7) | 1.10 (0.80, 1.53) | 0.96 (0.68, 1.36) | 0.806 |
| Elementary school or below | 815 (22.8) | 1.56 (1.30, 1.87) | 737 (22.2) | 2.27 (1.65, 3.15) | 1.30 (0.93, 1.84) | 0.124 |
| 2 optimal health behaviors | | | | | | |
| College or above | 382 (18.6) | 1.00 (ref.) | 208 (8.2) | 1.00 (ref.) | 1.00 (ref.) | / |
| High school | 492 (14.7) | 0.85 (0.72, 1.01) | 681 (7.7) | 1.05 (0.88, 1.26) | 1.21 (0.95, 1.54) | 0.120 |
| Middle school | 587 (13.2) | 0.80 (0.68, 0.94) | 984 (8.5) | 1.10 (0.92, 1.31) | 1.34 (1.06, 1.69) | 0.014 |
| Elementary school or below | 519 (19.4) | 1.37 (1.13, 1.65) | 1729 (15.3) | 1.91 (1.60, 2.29) | 1.42 (1.13, 1.80) | 0.003 |
| 3-4 optimal health behaviors | | | | | | |
| College or above | 201 (15.9) | 1.00 (ref.) | 161 (7.4) | 1.00 (ref.) | 1.00 (ref.) | / |
| High school | 179 (10.7) | 0.77 (0.60, 0.98) | 380 (5.3) | 0.90 (0.73, 1.12) | 1.15 (0.84, 1.58) | 0.399 |
| Middle school | 212 (12.1) | 0.86 (0.67, 1.10) | 521 (6.7) | 0.98 (0.79, 1.20) | 1.10 (0.81, 1.49) | 0.549 |
| Elementary school or below | 166 (18.6) | 1.67 (1.24, 2.24) | 653 (11.2) | 1.72 (1.38, 2.15) | 1.10 (0.80, 1.52) | 0.545 |

CKM, cardiovascular-kidney-metabolic; LE8, Life’s Essential 8; OR, odds ratio; CI, confidential interval; ROR, ratio of odds ratio.

Analyses were conducted in participants with complete information on LE8 health behaviors (N=99124). Models were adjusted for age, study center, other socioeconomic factors including marital status, living status and occupation, as well as medication information including hypoglycemic drugs, antihypertensive drugs and lipid-lowering drugs (use or not). Multiplicative interaction was evaluated using OR for the product term between LE8 health behavior (0-1 optimal health behavior versus 3-4 optimal health behaviors) and education level (elementary school or below versus college or above), and the multiplicative interaction was statistically significant when its CI does not include 1. Additive interaction was evaluated using relative excess risk due to interaction (RERI) between the LE8 health behavior and education level, and the additive interaction was statistically significant when its CI did not include 0. In each health behavior subgroup, those with highest education (college or above) were selected as the control group. In the total population, the interaction term between education and sex was added to obtain P for interaction and the women-to-men ratio of odds ratio (ROR).

**Multimedia Appendix 1 Table S9. Sensitivity Analysis: Joint associations of education level and LE8** **health behavior with high-risk CKM syndrome (stage 3 and 4) in men and women**

|  | **Men** | | **Women** | | **P _for interaction_** |
| --- | --- | --- | --- | --- | --- |
|  | **Cases/Total (%)** | **OR (95% CI)** | **Cases/Total (%)** | **OR (95% CI)** |  |
| College or above, 3-4 optimal health behaviors | 201 (15.9) | 1.00 (ref.) | 161 (7.4) | 1.00 (ref.) | / |
| College or above, 2 optimal health behaviors | 382 (18.6) | 1.12 (0.91, 1.39) | 208 (8.2) | 0.96 (0.76, 1.22) | 0.564 |
| College or above, 0-1 optimal health behavior | 350 (17.5) | 1.07 (0.87, 1.33) | 68 (11.3) | 1.13 (0.81, 1.58) | 0.460 |
| High school, 3-4 optimal health behaviors | 179 (10.7) | 0.77 (0.61, 0.98) | 380 (5.3) | 0.93 (0.75, 1.14) | 0.359 |
| High school, 2 optimal health behaviors | 492 (14.7) | 1.00 (0.82, 1.23) | 681 (7.7) | 1.08 (0.89, 1.31) | 0.377 |
| High school, 0-1 optimal health behavior | 596 (16.0) | 1.23 (1.01, 1.50) | 211 (9.8) | 1.21 (0.96, 1.54) | 0.706 |
| Middle school, 3-4 optimal health behaviors | 212 (12.1) | 0.80 (0.64, 1.02) | 521 (6.7) | 0.94 (0.77, 1.15) | 0.499 |
| Middle school, 2 optimal health behaviors | 587 (13.2) | 0.90 (0.74, 1.10) | 984 (8.5) | 1.06 (0.87, 1.28) | 0.177 |
| Middle school, 0-1 optimal health behavior | 892 (15.9) | 1.18 (0.98, 1.43) | 307 (10.7) | 1.23 (0.99, 1.54) | 0.531 |
| Elementary school or below, 3-4 optimal health behaviors | 166 (18.6) | 1.28 (0.99, 1.65) | 653 (11.2) | 1.41 (1.15, 1.73) | 0.722 |
| Elementary school or below, 2 optimal health behaviors | 519 (19.4) | 1.40 (1.14, 1.72) | 1729 (15.3) | 1.75 (1.45, 2.12) | 0.096 |
| Elementary school or below, 0-1 optimal health behavior | 815 (22.8) | 1.72 (1.41, 2.1) | 737 (22.2) | 2.43 (1.98, 2.99) | 0.007 |

CKM, cardiovascular-kidney-metabolic; LE8, Life’s Essential 8; OR, odds ratio; CI, confidential interval; ROR, ratio of odds ratio.

Analyses were conducted in participants with complete information on LE8 health behaviors (N=99124). Models were adjusted for age, study center, and other socioeconomic factors including marital status, living status and occupation, as well as medication information including hypoglycemic drugs, antihypertensive drugs and lipid-lowering drugs (use or not). The combination of highest education (college or above) and 3-4 optimal health behaviors was selected as the control group. In the total population, the P for interaction was obtained by the product term of combined education-behavior group (Elementary school or below & 0-1 optimal health behavior versus college or above & 3-4 optimal health behaviors) and sex.

**Multimedia Appendix 1 Table S10. Sensitivity Analysis: Sex-specific odds ratios (and 95% CIs) and women-to-men ratio of odds ratios for moderate-risk and high-risk CKM syndrome attributable to low education in multiple imputation data sets imputed for missing baseline information and outcome**

|  | **Cases/Total**  **(%)** | **MODEL 1** | | | | **MODEL 2** | | | |
| --- | --- | --- | --- | --- | --- | --- | --- | --- | --- |
|  |  | **Men**  **OR (95% CI)** | **Women**  **OR (95% CI)** | **Women-to-men ROR** | **P _for interaction_** | **Men**  **OR (95% CI)** | **Women**  **OR (95% CI)** | **Women-to-men ROR** | **P _for interaction_** |
| OR for the prevalence of moderate-risk CKM syndrome | | | | | | | | | |
| High education | 151864 (84.0) | 1.00 (ref.) | 1.00 (ref.) | / | / | 1.00 (ref.) | 1.00 (ref.) | / | / |
| Low education | 62011 (78.6) | 0.91 (0.82, 1.001) | **1.38 (1.29, 1.47)** | **2.33 (2.10, 2.59)** | <0.0001 | 0.93 (0.84, 1.02) | **1.39 (1.30, 1.49)** | **2.27 (2.04, 2.51)** | <0.0001 |
| OR for the prevalence of high-risk CKM syndrome | | | | | | | | | |
| High education | 20576 (11.4) | 1.00 (ref.) | 1.00 (ref.) | / | / | 1.00 (ref.) | 1.00 (ref.) | / | / |
| Low education | 14484 (18.4) | **1.23 (1.17, 1.30)** | **1.26 (1.21, 1.31)** | 1.00 (0.94, 1.05) | 0.875 | **1.30 (1.23, 1.37)** | **1.29 (1.24, 1.35)** | 0.95 (0.90, 1.01) | 0.095 |

CKM, cardiovascular-kidney-metabolic; OR, odds ratio; CI, confidential interval; ROR, ratio of odds ratio.

Missing data for missing information were imputed. The number (percentage) of missing data was 6167 (2.38%) for educational attainment, 4660 (1.79%) for marital status, 5501 (2.12%) for living arrangements, 9036 (3.48%) for tobacco use, 30879 (11.89%) for sleep duration, 19998 (7.7%) for physical activity, 59771 (23.02%) for diet score, 5385 (2.07%) for BMI, 8972 (3.46%) for waist circumference, 2256 (0.87%) for systolic blood pressure, 2309 (0.89%) for diastolic blood pressure, 4087 (1.57%) for fasting glucose, 9404 (3.62%) for 2-hour post-load glucose, 2251 (0.87%) of HbA1c, 105374 (40.58%) for creatinine, 105374 (40.58%) for urinary albumin-creatinine ratio, 1078 (0.42%) for low-density lipoprotein cholesterol, 1060 (0.41%) for high-density lipoprotein cholesterol, 1258 (0.48%) for TG, 998 (0.38%) for total cholesterol, 18920 (7.29%) for CVD. We employed multiple imputation based on fully conditional specification (FCS) with arbitrary missing patterns to correct for response bias under the assumption of missing at random, and to maximally utilize existing risk factor data.

Model 1 was adjusted for age, study center and other socioeconomic factors including marital status, living status and occupation. Model 2 was further adjusted for Life’s Essential 8 health behavior including nicotine exposure, physical activity, diet and sleep, as well as medication information including hypoglycemic drugs, antihypertensive drugs and lipid-lowering drugs (use or not). For high-risk CKM syndrome, CKM stages 0 to 2 were used as reference; for moderate-risk CKM syndrome, CKM stages 0 was used as reference. Odds ratio was calculated by contrasting low education (primary school or below) with high education (middle school or above), in men and women, respectively. In total population, the interaction term between education and sex was added to obtain P for interaction and the women-to-men ratio of odds ratio (ROR).

**Multimedia Appendix 1 Table S11. Sensitivity Analysis: Achievement of optimal LE8 health behavior and health factor targets in men and women with different education levels, according to different CKM stages in multiple imputation data sets imputed for missing baseline information and outcome**

|  | **No/Total (%)** | **Optimal health behavior** | **No/Total (%)** | **Optimal health factors** |
| --- | --- | --- | --- | --- |
| **All participants** |  |  |  |  |
| Men, high education | 5537/69677 (7.9) | 8.27 (8.05, 8.50) | 10947/69677 (15.7) | 11.99 (11.74, 12.24) |
| Men, low education | 1205/20352 (5.9) | 6.32 (5.98, 6.69) | 3576/20352 (17.6) | 17.84 (17.28, 18.42) |
| Women, high education | 14783/111111 (13.3) | 13.67 (13.43, 13.92) | 27290/111111 (24.6) | 17.81 (17.55, 18.07) |
| Women, low education | 6327/58517 (10.8) | 11.4 (11.11, 11.69) | 9073/58517 (15.5) | 14.52 (14.21, 14.83) |
| **CKM syndrome Stage 0** |  |  |  |  |
| Men, high education | 194/2276 (8.5) | 8.39 (7.24, 9.71) | 2024/2276 (88.9) | 89.36 (87.80, 90.74) |
| Men, low education | 45/746 (6.0) | 5.90 (4.37, 7.92) | 667/746 (89.4) | 91.34 (89.04, 93.19) |
| Women, high education | 888/6072 (14.6) | 14.45 (13.36, 15.62) | 5597/6072 (92.2) | 91.06 (90.09, 91.94) |
| Women, low education | 233/1628 (14.3) | 14.07 (12.27, 16.08) | 1475/1628 (90.6) | 91.16 (89.49, 92.58) |
| **CKM syndrome Stage 1** |  |  |  |  |
| Men, high education | 1120/12681 (8.8) | 9.23 (8.68, 9.80) | 4935/12681 (38.9) | 35.99 (35.06, 36.93) |
| Men, low education | 233/3804 (6.1) | 6.46 (5.68, 7.34) | 1597/3804 (42.0) | 42.16 (40.49, 43.85) |
| Women, high education | 4166/28686 (14.5) | 15.00 (14.49, 15.51) | 13923/28686 (48.5) | 43.47 (42.79, 44.16) |
| Women, low education | 1317/10345 (12.7) | 13.31 (12.58, 14.08) | 4492/10345 (43.4) | 41.39 (40.33, 42.46) |
| **CKM syndrome Stage 2** |  |  |  |  |
| Men, high education | 3423/43388 (7.9) | 8.10 (7.82, 8.39) | 3415/43388 (7.9) | 5.73 (5.52, 5.96) |
| Men, low education | 692/10814 (6.4) | 6.51 (6.05, 7.01) | 900/10814 (8.3) | 7.54 (7.06, 8.05) |
| Women, high education | 8787/67109 (13.1) | 13.43 (13.13, 13.75) | 7193/67109 (10.7) | 8.07 (7.85, 8.31) |
| Women, low education | 4110/37048 (11.1) | 11.33 (10.97, 11.71) | 2747/37048 (7.4) | 6.80 (6.53, 7.08) |
| **CKM syndrome Stage 3** |  |  |  |  |
| Men, high education | 445/6325 (7.0) | 7.81 (7.08, 8.61) | 232/6325 (3.7) | 2.39 (2.02, 2.82) |
| Men, low education | 160/3523 (4.5) | 5.03 (4.29, 5.88) | 259/3523 (7.4) | 3.78 (3.19, 4.49) |
| Women, high education | 319/2844 (11.2) | 12.49 (11.18, 13.93) | 76/2844 (2.7) | 1.86 (1.44, 2.38) |
| Women, low education | 345/5109 (6.8) | 7.49 (6.70, 8.37) | 146/5109 (2.9) | 1.36 (1.11, 1.67) |
| **CKM syndrome Stage 4** |  |  |  |  |
| Men, high education | 355/5007 (7.1) | 7.96 (7.13, 8.88) | 341/5007 (6.8) | 5.63 (4.99, 6.34) |
| Men, low education | 75/1465 (5.1) | 5.72 (4.56, 7.16) | 153/1465 (10.4) | 10.04 (8.53, 11.78) |
| Women, high education | 623/6400 (9.7) | 10.89 (10.00, 11.85) | 501/6400 (7.8) | 6.24 (5.62, 6.91) |
| Women, low education | 322/4387 (7.3) | 8.15 (7.26, 9.12) | 213/4387 (4.9) | 4.43 (3.84, 5.11) |

CKM, cardiovascular-kidney-metabolic.

Age-adjusted proportions (and 95% confidential intervals) were obtained from logistic regressions. A restricted cubic spline function was applied to age with 4 knots placed at the 5th, 35th, 65th, and 95th percentiles.

**Multimedia Appendix 1 Table S12. Sensitivity Analysis: Association of low education with high-risk CKM syndrome (stage 3 and 4) in men and women with different LE8 health behavior in multiple imputation data sets imputed for missing baseline information and outcome**

|  | **Men** | | **Women** | | **Women-to-men**  **ROR** | **P _for interaction_** |
| --- | --- | --- | --- | --- | --- | --- |
|  | **Cases/Total (%)** | **OR (95% CI)** | **Cases/Total (%)** | **OR (95% CI)** |  |  |
| Multiplicative and additive interaction between health behavior and education level | OR for product term 1.09,  95% CI 0.95 to 1.24.  RERI 0.23, 95% CI 0.13 to 0.33 | | OR for product term 1.12,  95% CI 1.02 to 1.24.  RERI 0.39, 95% CI 0.32 to 0.46 | | / | / |
| 0-1 optimal health behavior | | | | | | |
| High education | 5396 (16.7) | 1.00 (ref.) | 1674 (10.4) | 1.00 (ref.) | / | / |
| Low education | 2711 (26.1) | **1.52 (1.42, 1.62)** | 2299 (20.3) | **1.82 (1.66, 1.99)** | **1.12 (1.02, 1.23)** | **0.020** |
| 2 optimal health behaviors | | | | | | |
| High education | 4296 (16.2) | 1.00 (ref.) | 5004 (8.4) | 1.00 (ref.) | 1.00 (ref.) | / |
| Low education | 1767 (23.1) | **1.65 (1.52, 1.79)** | 5377 (16.4) | **1.78 (1.68, 1.87)** | **1.10 (1.02, 1.20)** | **0.019** |
| 3-4 optimal health behaviors | | | | | | |
| High education | 1640 (15.0) | 1.00 (ref.) | 2566 (7.2) | 1.00 (ref.) | 1.00 (ref.) | / |
| Low education | 510 (21.8) | **1.83 (1.58, 2.11)** | 1820 (12.7) | **1.78 (1.64, 1.94)** | 1.00 (0.87, 1.15) | 0.994 |

CKM, cardiovascular-kidney-metabolic; LE8, Life’s Essential 8; OR, odds ratio; CI, confidential interval; ROR, ratio of odds ratio; RERI, relative excess risk due to interaction.

Models were adjusted for age, study center, other socioeconomic factors including marital status, living status and occupation, as well as medication information including hypoglycemic drugs, antihypertensive drugs and lipid-lowering drugs (use or not). In each health behavior subgroup, those with high education (middle school or above) were selected as the control group. Multiplicative interaction was evaluated using OR for the product term between LE8 health behavior (0-1 optimal health behavior versus 3-4 optimal health behaviors) and education level (low versus high), and the multiplicative interaction was statistically significant when its CI does not include 1. Additive interaction was evaluated using relative excess risk due to interaction (RERI) between the LE8 health behavior and education level, and the additive interaction was statistically significant when its CI did not include 0. In the total population, the interaction term between education and sex was added to obtain P for interaction and the women-to-men ratio of odds ratio (ROR).

**Multimedia Appendix 1 Table S13.** **Joint associations of education level and LE8 health behavior with high-risk CKM syndrome (stage 3 and 4) in men and women in multiple imputation data sets imputed for missing baseline information and outcome**

|  | **Men** | | **Women** | | **P _for interaction_** |
| --- | --- | --- | --- | --- | --- |
|  | **Cases/Total (%)** | **OR (95% CI)** | **Cases/Total (%)** | **OR (95% CI)** |  |
| High education, 3-4 optimal health behaviors | 1640 (15.0) | 1.00 (ref.) | 2566 (7.2) | 1.00 (ref.) | / |
| High education, 2 optimal health behaviors | 4296 (16.2) | 1.16 (1.09, 1.25) | 5004 (8.4) | 1.12 (1.06, 1.18) | 0.685 |
| High education, 0-1 optimal health behavior | 5396 (16.7) | 1.38 (1.29, 1.48) | 1674 (10.4) | 1.38 (1.29, 1.48) | 0.119 |
| Low education, 3-4 optimal health behaviors | 510 (21.8) | 1.51 (1.33, 1.72) | 1820 (12.7) | 1.55 (1.44, 1.66) | 0.996 |
| Low education, 2 optimal health behaviors | 1767 (23.1) | 1.75 (1.60, 1.91) | 5377 (16.4) | 1.88 (1.77, 2.00) | 0.067 |
| Low education, 0-1 optimal health behavior | 2711 (26.1) | 2.09 (1.93, 2.27) | 2299 (20.3) | 2.36 (2.20, 2.54) | 0.003 |

CKM, cardiovascular-kidney-metabolic; LE8, Life’s Essential 8; OR, odds ratio; CI, confidential interval; ROR, ratio of odds ratio.

Models were adjusted for age, study center, other socioeconomic factors including marital status, living status and occupation, as well as medication information including hypoglycemic drugs, antihypertensive drugs and lipid-lowering drugs (use or not). The combination of high education (middle school or above) and 3-4 optimal health behaviors was selected as the control group. In the total population, the P for interaction was obtained by the product term of combined education-behavior group (low education & 0-1 optimal health behavior versus high education & 3-4 optimal health behaviors) and sex.
